# Comparison of genes involved in brain development: insights into the organization and evolution of the telencephalic pallium

**DOI:** 10.1038/s41598-024-51964-1

**Published:** 2024-03-13

**Authors:** Jiangyan Zhang, Rui Zhao, Shiying Lin, Dong Yang, Shan Lu, Zenan Liu, Yuanyuan Gao, Yiyun Zhang, Bing Hou, Chao Xi, Jin Liu, Jie Bing, Erli Pang, Kui Lin, Shaoju Zeng

**Affiliations:** 1 Beijing Key Laboratory of Gene Resource and Molecular Development, Beijing Normal University, Beijing, China; 2 Beijing Key Laboratory of Genetic Engineering Drugs and Biological Technology, Beijing Normal University, Beijing, China; 3 MOE Key Laboratory for Biodiversity Science and Ecological Engineering, College of Life Sciences, Beijing Normal University, Beijing, China

**Keywords:** Evolutionary biology, Neurogenesis

## Abstract

The mechanisms underlying the organization and evolution of the telencephalic pallium are not yet clear.. To address this issue, we first performed comparative analysis of genes critical for the development of the pallium (*Emx1/2* and *Pax6*) and subpallium (*Dlx2* and *Nkx1/2*) among 500 vertebrate species. We found that these genes have no obvious variations in chromosomal duplication/loss, gene locus synteny or Darwinian selection. However, there is an additional fragment of approximately 20 amino acids in mammalian Emx1 and a poly-(Ala)_6–7_ in Emx2. Lentiviruses expressing mouse or chick *Emx2* (*m-Emx2* or *c-Emx2* Lv) were injected into the ventricle of the chick telencephalon at embryonic Day 3 (E3), and the embryos were allowed to develop to E12–14 or to posthatchling. After transfection with *m-Emx2* Lv, the cells expressing Reelin, Vimentin or GABA increased, and neurogenesis of calbindin cells changed towards the mammalian inside-out pattern in the dorsal pallium and mesopallium. In addition, a behavior test for posthatched chicks indicated that the passive avoidance ratio increased significantly. The study suggests that the acquisition of an additional fragment in mammalian Emx2 is associated with the organization and evolution of the mammalian pallium.

## Introduction

The brain, the most complex and fascinating organ, is a focal point for studying development and evolution^1^. The pallium, especially the dorsal pallium, largely differs in each lineage of vertebrates: it is absent as a morphological entity in amphioxus^2^ and is present as only a layered area in fishes and amphibians, while it is a simply layered region in reptiles and aves, but a large intricate multilayered cortex in mammals^3^. In contrast, the subpallial regions or the subpallium, the deep-seated basal ganglia (striatum and pallidum), are highly conserved in vertebrates with respect to the expression patterns of transcription factors, neuronal types and some neural connections, despite varieties in shapes and sizes^4–6^. Thus, from an evolutionarily point of view, the pallium has undergone divergent trajectories in different vertebrates, and the dorsal pallium has experienced the greatest development in mammals^7,8^. To date, the mechanism is not yet well known for the organization and evolution of the telencephalic pallium.

Comparative molecular studies indicate that some homeobox-containing genes are essential for defining the discrete regions of the vertebrate telencephalon and for specifying the identity or activity of cells in each subregion^9,10^. These genes display conserved nested expression in the telencephalon of the examined species (such as frog, turtle, chick, and mouse). In particular, *Pax6* is expressed throughout the pallium, whereas *Emx1* and *Emx2* are expressed in the dorsal or lateral pallium, and *Dlx1/2* and *Nkx2.1* are only expressed in the striatum and pallidum, respectively^11,12^. Mutant mice for these genes exhibit developmental defects in their expression regions, some of which display characteristics that are very similar to those of the nonmammalian telencephalon, including poor cortical lamination, a lack of the corpus callosum, and fewer cells expressing Reelin^13–19^, which guides neural progenitor cells to migrate along radial glial fibers in the deep-to-superficial direction during pallium development^20,21^. However, it remains unknown whether the evolution of the mammalian pallium is concerned with the changes in the above genes, although some reports indicate that brain development is affected by some gene changes, including duplication (gene, chromosome or even genome)^22–24^, insertion or deletion^25^, single or multiple mutations^26^, and Darwinian selection^27^.

To address the above issue, we first performed comparative analysis of the homeobox genes involved in the development of the pallium (*Pax6* and *Emx1/2*) and the subpallium (*Dlx2* and *Nkx1/2*) using the currently available data from over 500 species, covering all the major lineages of chordates. Our analysis indicated that each of the studied genes displayed no obvious variations in chromosomal duplication/loss, gene locus synteny, gene sequence or Darwinian selection, except that there was an additional fragment of approximately 20 amino acids or a poly-(Ala)_6–7_ sequence in Emx1 and Emx2, respectively, in all of the examined mammals (188 species). After the injection of lentiviruses expressing mouse or chick *Emx2* into the ventricles of chick telencephalons at E3 (embryonic Day 3), we examined and compared the changes of cells expressing Reelin, Vimentin or GABA, and the patterns of cell migration in the dorsal pallium (Wulst, W) and the other two parts of pallium: mesopallium (M) and nidopallium (N), which molecularly and structurally belong to the lateral and ventral pallium, respectively^11,28–31^. The study suggests that the acquisition of an additional fragment in mammalian Emx2 may be related to the organization and evolution of the mammalian pallium.

## Results

### Bioinformatic analysis of the evolution of the studied genes

#### Gene duplication/loss, sequence alignment and gene synteny

To identify gene duplication/loss, we analyzed the chromosomal loci of the studied homeobox genes involved in the development of the pallium (*Pax6* and *Emx1/2*) and the subpallium (*Dlx2* and *Nkx1/2*). Among the examined species (Table S1), in which the chromosomal loci of these genes are open for inquiry, nearly all of these genes are present at a single chromosomal locus with a single copy, except that 1) there is one *Emx* gene in the ascidian class, but two, i.e., *Emx1* (or *Emx A*) and *Emx2* (or *Emx B*), in vertebrates, while in some fish, amphibian or marsupial species, there is an additional *Emx3*; 2) both *Pax6a* and *Pax6b* are present in zebrafish but only *Pax6* in other vertebrate species.

We then compared the protein sequences encoded by the examined genes between the examined species. The results indicated that Emx1 acquired an additional fragment of ~ 20 amino acids in length and that Emx2 acquired an additional tract of poly-(Ala)_6–8_ in all 188 examined mammalian species compared to nonmammals (300 species), except that Emx2 contained only poly-(Ala)_2_ in the examined monotreme (duckbill platypus) and marsupials (gray short-tailed opossum and Tasmanian devil) (Figs. S1 and S2), in which the corpus callosum is lacking, as in *Emx2*-deficient mice^16^, but not in Eutheria. Similar patterns were not found in other regions of *Emx1/2* or in the other examined genes (Figs. S3–S5).

**Figure 1:**
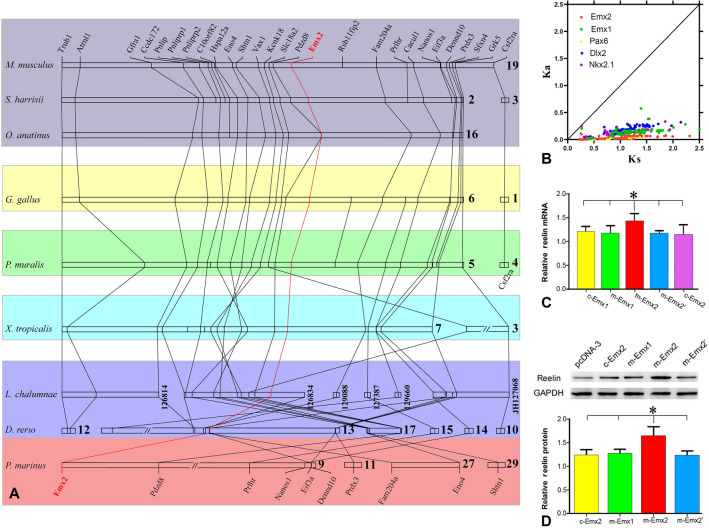
Synteny of *Emx2* and its neighboring genes, Ka/Ks ratios of studied genes and the expression levels of Reelin mRNA and protein. (**A**) Synteny of *Emx2* (red) and its neighboring genes within 3.8 Mb chromosome (Chr) length. Numbers to the right of bars indicate Chr numbers. The compared species include monotreme (*Ornithorhynchus anatinus*), marsupial (*Sarcophilus harrisii*), chick (*Gallus gallus*), gecko (*Podarcis muralis*), Xenopus (*Xenopus tropicalis*), Latimeria (*Latimeria chalumnae*), zebrafish (*Danio rerio*) and lamprey (*Petromyzon marinus*). The chromosome localization maps were drawn by MapChart 2.32. (**B**) Ka/Ks ratios of studied genes among some representative species of vertebrates by using KaKs_Calculator 2.0. The species include 20 mammals, 20 birds, 20 reptiles, 7 amphibians, 24 fishes (1 Coelacanthiforme, 20 Actinopterygii and 3 Chondrichthyes), and 1 Petromyzontiformes. The Latin names of these species are listed in Table S3. C and D: The mRNA (**C**) and protein (**D**) expression levels of Reelin normalized to those of GAPDH after transfection with the expression plasmid for mouse *Emx2* (*m-Emx2*), chicken *Emx1*(c-Emx1) or *Emx2*(*c-Emx2*), mouse *Emx1* (*m-Emx1*) or assumed mouse Emx2 with a tract of 5 alanine residues (*m-Emx2’*), relative to pcDNA-3 (control plasmids containing no studied gene). The belts of Reelin or GAPDH were from the same gels. The data are presented as the mean ± a standard error (SEM). An asterisk indicates a significant increase after transfection with the expression plasmid for mouse Emx2 compared to the other plasmids (SPSS, one-way ANOVA, *p *< 0.05).

We next compared the synteny of the examined homeobox genes in the chromosomes (Chrs) to gain more evolutionary aspects. For simplicity, thirty genes adjacent to the examined genes of mouse (Chr length ranged from 2.7 to 6.8 Mb) were compared only in some representative vertebrates (shown in Table S2). For the synteny of mouse *Emx2* on Chr 19, all thirty genes were located on one chromosome except *Csf2ra* in other amniote species (Fig. 1A). However, these genes are distributed on chromosomes 4–6 in fish (*Latimeria chalumnae* or *Danio rerio*) and cyclostome (*Lethenteron japonicum*). For the other examined genes, the results are shown in Table S2 and Figs. S6–S9. Similar to *Emx2,* the synteny in the fish and cyclostome in the chromosomes indicated large differences between them and tetrapods.

#### Phylogenetic tree and adaptive evolution

To further understand the evolution of the examined homeobox genes, molecular phylogenetic trees of the encoded protein sequences were constructed (Figs. S10–S14). Phylogenetic trees indicated that species in the major lineages of vertebrates (fish, amphibians, reptiles, aves, and mammals) were generally clustered into the same branch. However, reptile species were grouped into more branches, in contrast to aves and mammals.

To understand the Darwinian selection of these homeobox genes, we calculated the Ka/Ks ratios for some representative species of vertebrates (Fig. 1B). The Ka/Ks ratios between the species of all of the genes were < 1, suggesting a deviation from neutral expectations (Ka/Ks = 1) and negative Darwinian selection during the evolution of vertebrates (one-tailed z-tests, *p *< 0.05).

#### Tertiary structure prediction

As no homology could be detected between mouse Emx2 and any other solved protein structures in the RCSB protein data bank, except for the homeodomain located between loci 157 and 213, a de novo protein structure prediction was calculated. By using Rosetta solutions, a helix was predicted, aside from the homeodomain, which contained a tract of poly-(Ala)_7_ in mouse or poly-(Ala)_6_ in human Emx2 (Fig. S15A and B). Using another calculation method (Dompred), a helix was also obtained for poly-(Ala)_7_ in mouse Emx2 but not in mouse Emx1 (Fig. S16) or chick Emx 1 or 2 (Fig. S17). However, a loop, not a helix, was predicted, assuming a poly-(Ala)_5_ in mouse or human Emx2 (not shown), suggesting that at least six polyalanine repeats are necessary to form a helix.

### Transferring m-Emx1/2 into cells from chicken embryonic brains and its effects on Reelin expression

To assess the possible role of an additional fragment present in mammalian Emx1 and Emx2, we compared the mRNA expression levels of Reelin, which has been shown to be decreased or absent in mutant *Emx2* mice^14,15^, in cultured cells isolated from the embryonic telencephalons (Embryonic day 8, E8) of chicks following transfection with pcDNA-3 plasmids expressing mouse or chicken *Emx1*/*2*. Our results showed that the Reelin mRNA expression levels were significantly increased after transfection with the expression plasmids for mouse *Emx2*, in contrast to those for mouse *Emx1* or chicken *Emx1*/*Emx2* (Fig. 1C). However, this increase did not appear if the number of poly-Ala residues was reduced from 7 to 5 in mouse Emx2, i.e., poly-(Ala)_7_ → (Ala)_5_ (Fig. 1C, mouse Emx2). Based on Western blot analysis, we found that the expression of the Reelin protein was significantly increased in cultured cells transfected with the expression plasmid for mouse *Emx2* compared to those for chick *Emx2*, mouse *Emx1* or mouse *Emx2* containing a reduced number of poly-Ala residues, poly-(Ala)_5_ (n = 5) (Fig. 1D).

### Transferring m-Emx2 into chicken embryonic brains and its validation

#### Lentivirus infection

**Figure 2:**
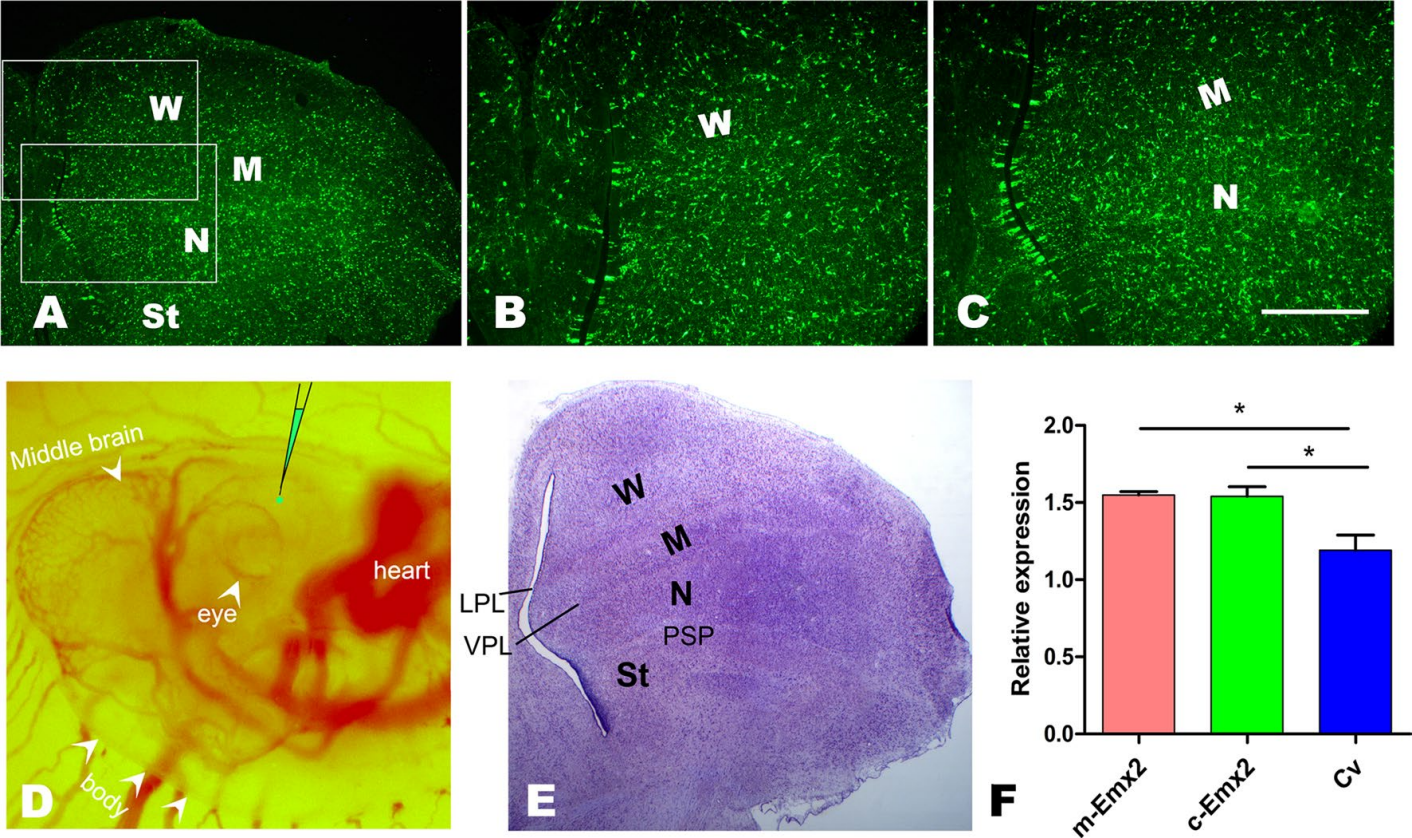
Green fluorescent protein (GFP) expression in chick embryo telencephalon at embryonic day (E) 16 infected with lentiviruses at E3. (**A**) Cells infected with lentiviruses expressing GFP fluorescence were uniformly distributed in Wulst (W), Mesopallium (M), Nidopallium (N) and Striatum (St). (**B** and **C**) Amplification of the boxed areas in A. (**D**) The injection site of lentiviruses into the ventricle of the telencephalon at E3. The ventricle site can be determined with reference to the developing heart, eye and midbrain. Nissl staining of the brain section corresponding to A. Scale bars: 150 µm for B and C, 400 µm for A and E, and 1.5 mm for D. (**F**) Expression levels of *m-Emx2* and *c-Emx2* mRNA in the chick telencephalon after infection with control lentiviruses (Cv) and lentiviruses expressing mouse *Emx2* (*m-Emx2*) and chick *Emx2* (*c-Emx2*). The data are presented as the mean ± a standard error (SEM). *p < 0.05. LPL: lateral pallial lamina, VPL: ventral pallial lamina, PSP: pallial-subpallial boundary.

To evaluate the role of the additional fragment in mammalian *Emx2* in brain development, lentiviruses expressing mouse *Emx2* were injected into the ventricles of the telencephalons in chicks at E3. The infected cells expressing GFP fluorescence were distributed in W, M, N and St, and they were approximately homogenously located in each of the above regions, as observed on E12 (Fig. 2A–C,E). The distribution of the infected cells expressing GFP fluorescence was not obviously different between the group receiving an injection of control lentiviruses (Cv, without insertion of any target gene) and the group receiving an injection of the lentiviruses expressing mouse or chick Emx2. Few cells infected with lentiviruses were distributed in the outside of the telencephalon (not shown). This indicates that the chick telencephalon was successfully infected after an injection of lentiviruses into the ventricle of the telencephalon at E3 (Fig. 2D).

**Figure 3:**
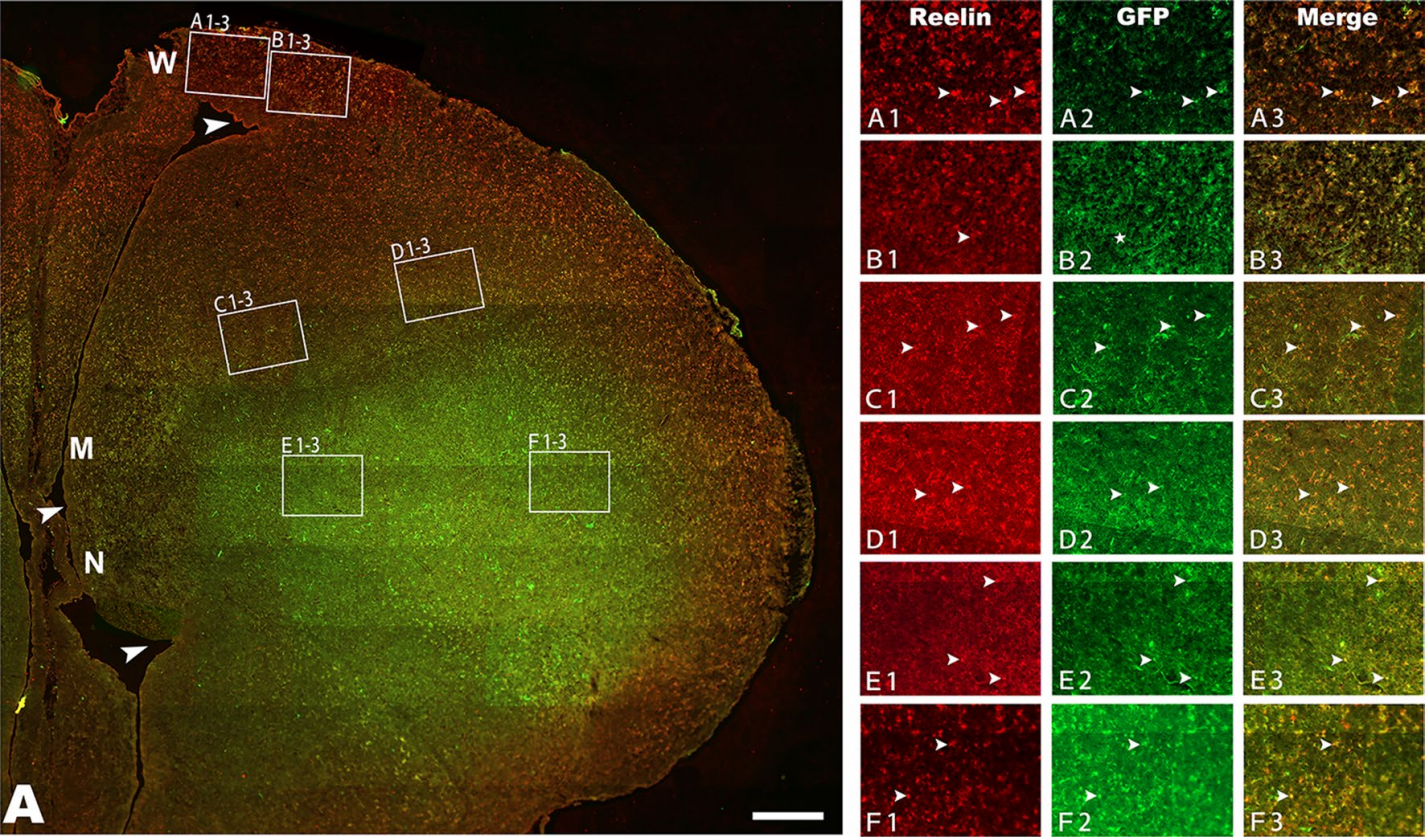
Reelin expression in the pallium at embryonic day (E) 16. Reelin expression in the Wulst (W), Mesopallium (M) and Nidopallium (N) after injection of lentiviruses expressing mouse Emx2 (*m-Emx2*) into the ventricle of the telencephalon at E3. The boundaries of W, M and N are indicated by arrowheads. Reelin expression in medial (**A1–3**, **C1–3** and **E1–3**) and lateral (**B1–3**, **D1–3** and **F1–3**) areas is amplified in A1-F3, respectively. Some cells labeled for Reelin (red) and infected with lentiviruses (expressing GFP) are indicated by arrowheads. Scale bar in A = 200 µm.

We then examined and compared the expression of mouse or chick Emx2 in the chick telencephalon at E16 in the studied groups. The result indicated that mouse or chick Emx2 mRNA increased significantly after injection of lentiviruses encoding mouse or chick Emx2 compared to the chicks after the injection of Cv (Fig. 2F). In addition, there were no significant differences in the size and structure of the brains injected with Cv compared to the brains without injection, showing that the injection of lentiviruses into the ventricle of the telencephalon at E3 did not affect the normal development of the brain. However, the numbers of cells expressing GFP decreased from E12 to E16 (Figs. 3, 5 and SS17–S20).

#### Changes in immunohistochemical reactivities

**Figure 4:**
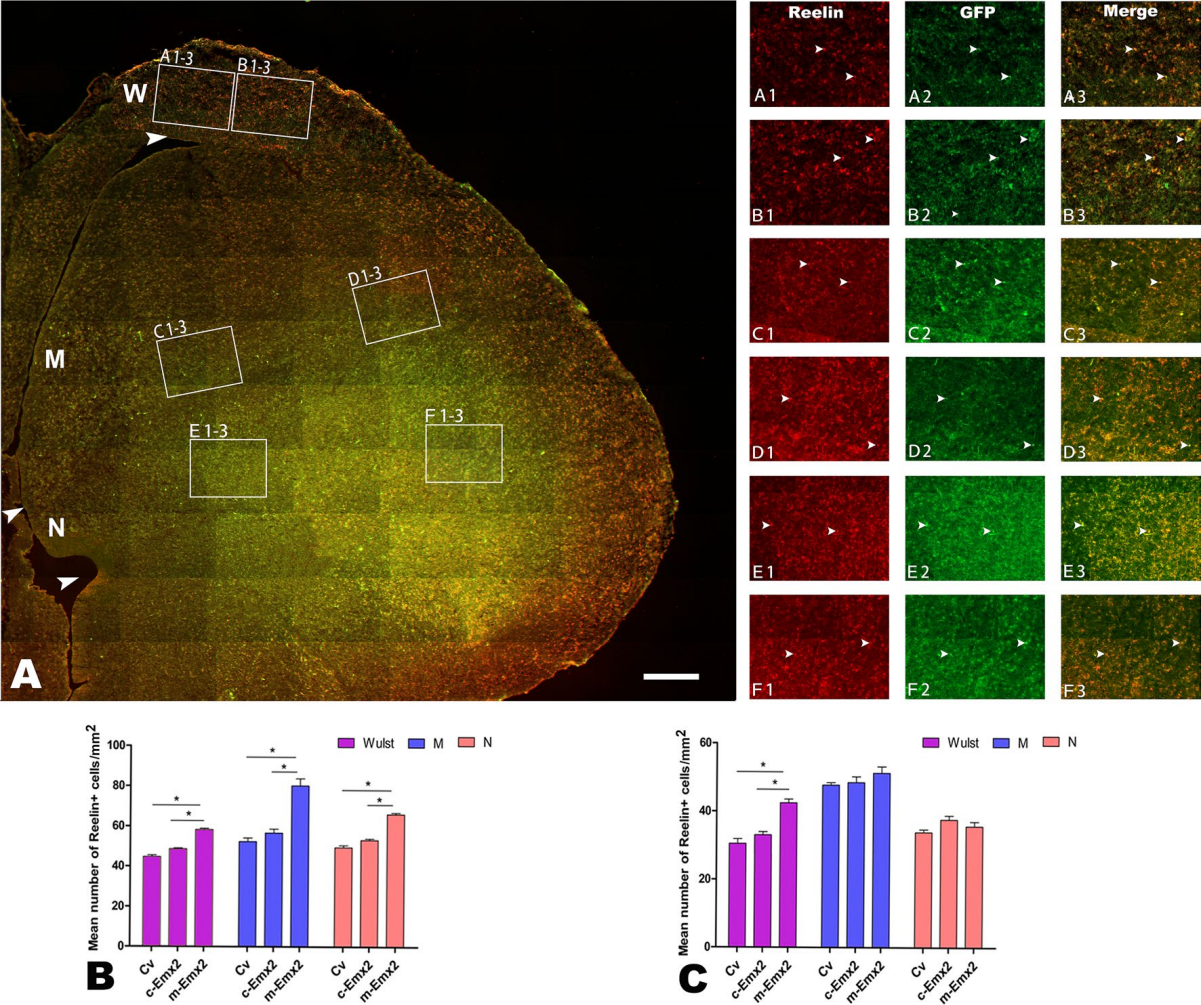
Reelin expression in the pallium at embryonic day (E) 16 after injection of lentiviruses expressing chick *Emx2 (c-Emx2*) into the ventricle of the telencephalon at E3. The boundaries of Wulst (W), Mesopallium (M) and Nidopallium (N) are indicated by arrowheads. (**A)** Reelin expression in medial (**A1–3**, **C1–3** and **E1–3**) and lateral (**B1–3**, **D1–3** and **F1–3**) areas is amplified in A1-F3, respectively. Some cells labeled for Reelin (red) and infected with lentiviruses (expressing GFP) are indicated by arrowheads. (**B** and **C)** Comparison of the numbers of Reelin cells per mm2 in the medial (**B**) and lateral (**C**) regions of W, M and N among the groups after injections of control lentiviruses (Cv) and *m-Emx2* or *c-Emx2* lentiviruses. Scale bar in A = 200 µm. The data are presented as the mean ± a standard error (SEM). *p < 0.05 (SPSS, one-way ANOVA).

**Figure 5:**
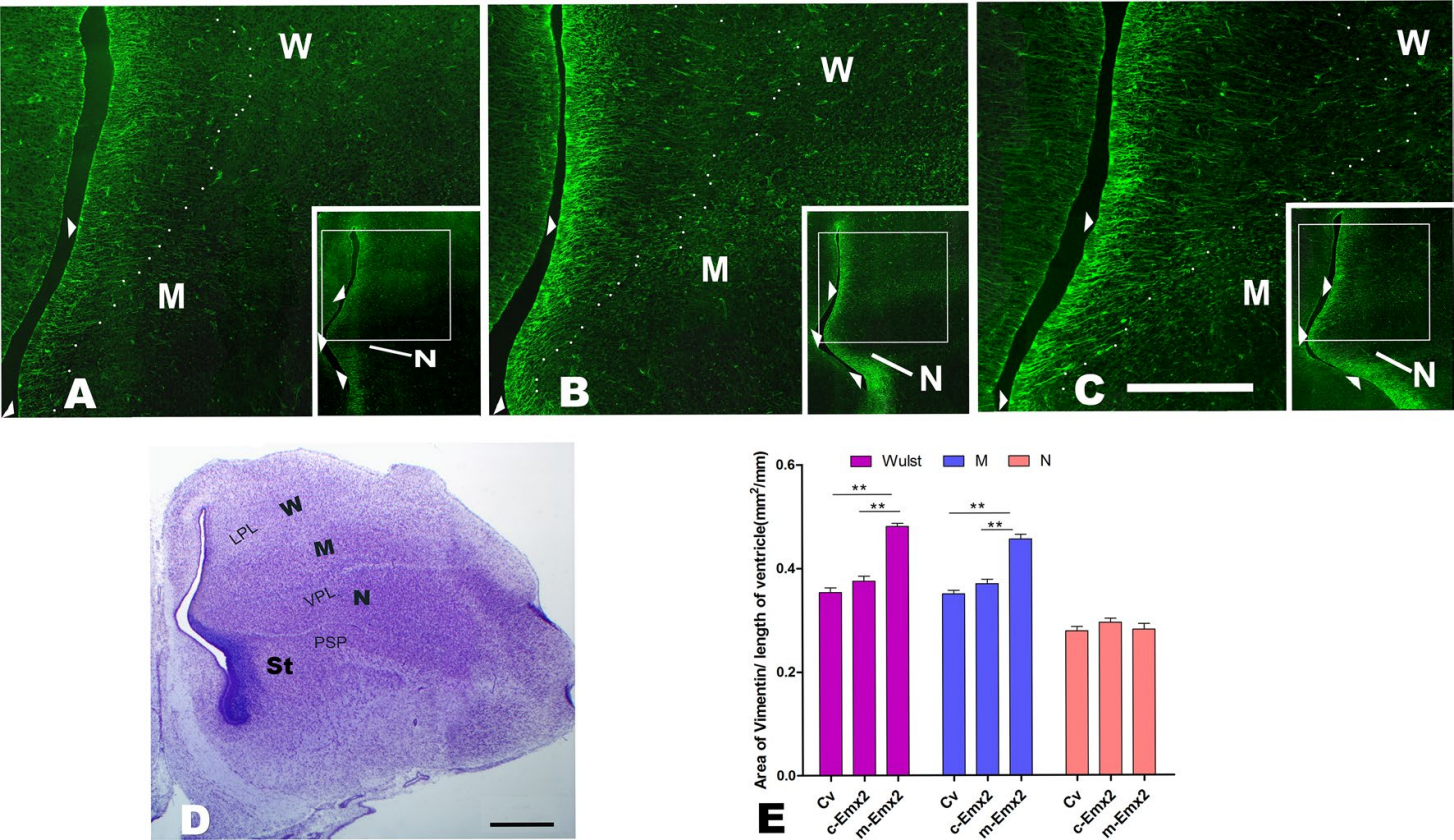
Vimentin expression in the pallium at embryonic day (E) 12. (**A** and **C**) Vimentin expression in the Wulst (W), Mesopallium (M) and Nidopallium (N) after injection of control lentiviruses (Cv, A) and lentiviruses expressing chick *Emx2 (c-Emx2*, **B**) or mouse *Emx2* (*m-Emx2*, **C**) into the ventricle of the telencephalon at E3. The small boxed areas in A-C are further amplified. Scale bar in C = 1.0 mm for the inserts in (**A**–**C**) and 300 µm for the amplified regions. (**D**) Nissl staining of brain sections corresponding to A-C. Bar = 500 µm. LPL: lateral pallial lamina, VPL: ventral pallial lamina, PSP: pallial-subpallial boundary. The ends of the fibers extending outwards are labeled with small dots, and the extending fibers could be traced continuously at least 100 μm long. (**E**) The areas shaped by these labeled dots and the ventricle (mm^2^) to the length of the ventricle zone (mm) were compared among the groups after injections of control lentiviruses (Cv) and *m-Emx2* or *c-Emx2* lentiviruses. The data are presented as the mean ± a standard error (SEM). **p < 0.01 (SPSS, one-way ANOVA).

**Figure 6:**
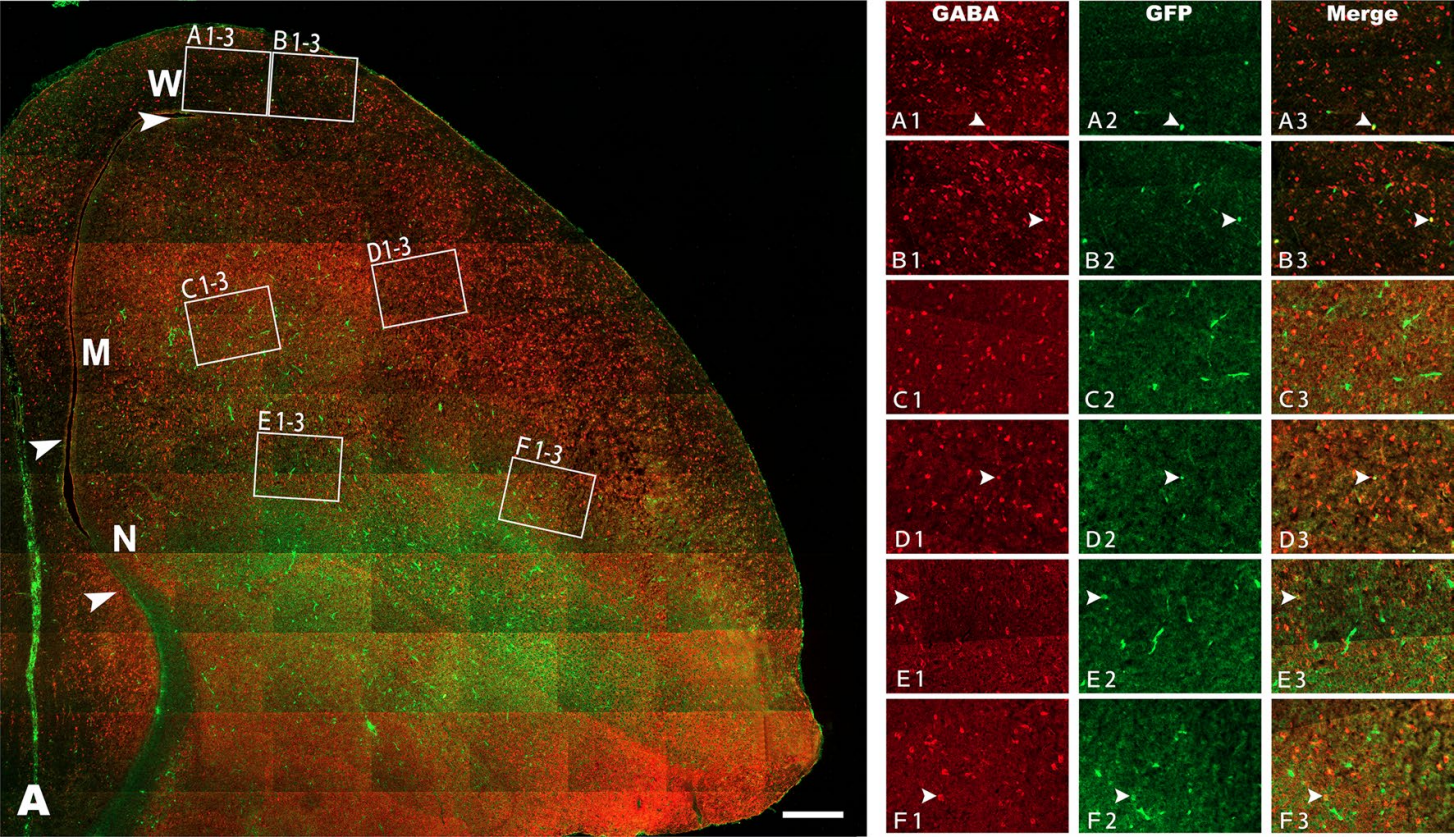
GABA distribution in the pallium at embryonic day (E) 16. GABA expression in the Wulst (W), Mesopallium (M) and Nidopallium (N) after injection of lentiviruses expressing mouse *Emx2* (*m-Emx2*) into the ventricle of the telencephalon at E3. The boundaries of W, M and N are indicated by arrowheads. GABA expression in medial or inside (**A1**–**3**, **C1**–**3** and **E1**–**3**) and lateral or outside (**B1**–**3**, **D1**–**3** and **F1**–**3**) areas is amplified in **A1**–**F3**, respectively. Some cells labeled for GABA (red) and infected with lentiviruses (expressing GFP) are indicated by arrowheads. Scale bar = 200 µm.

To examine the changes in immunohistochemical reactivities in the W, M and N, the brain level (approximately A 8.2 in the chick brain atlas^32^) that contains the above regions was chosen. We also examined and compared the other two brain levels rostrally (approximately A 10.2) or caudally (approximately A 6.2). In addition, the medial (inside) and lateral (outside) areas of each studied region (W, M and N) were examined at each brain level. The medial regions of W, M and N are located approximately 1/4 of the distance from the ventricle, whereas the lateral regions are located approximately 3/4 of the distance from the ventricle to the most outside of the brain, with each region occupying at least 400*600 μm^2^. Some representative regions are shown in Figs. 3, 4, 5, 6, and Figs. S17–S20. The results reported in the present study did not differ notably among the above examined brain levels (A6.2 to A10.2, not shown).

As previous reports have been shown that Reelin-positive Cajal-Retzius cells are lost and tangential migration of cells from the ganglionic eminence (such as GABA) into the neocortex is greatly inhibited^16^, while the laminar structures of pallium (including those containing MAP2 cells) are abnormal in *Emx2*-deficient mice^14–17^, immunohistochemical activities for Reelin, Vimentin, GABA and MAP2 were examined. Following the injection of *m-Emx2* Lv into the ventricle of the telencephalon at E3, cells expressing Reelin were increased significantly in the medial parts of W, M and N at E16*,* in contrast to the groups injected with *c-Emx2* Lv or Cv*.* However, these significant changes only appeared in the inside part of W but not in M and N at E16 (Figs. 3 and 4). Although there were slight increases in the numbers of Reelin-expressing cells after injection of *c-Emx2* Lv, they were not significant compared to the groups injected with Cv (Fig. 4B,C).

Based on previous reports, Reelin is correlated with Vimentin which guides cell migration^33,34^, and is expressed in radial glia^34,35^, and we thus examined the expression of Vimentin after injections of *m-Emx2* Lv*, c-Emx2* Lv or Cv*.* Vimentin-labeled fibers were seen to start from the ventricle (Fig. 5A–C). After the Vimentin fibers left the ventricle, they were found to continue extending outwards in W, but were relatively short and scarcely seen in N and were intermediately noted in M. The distribution of Vimentin fibers thus varied in W, M and N (Fig. 5A–E). We traced Vimentin fibers extending outwards as far as possible in W, M and N. To quantitively assess the distribution of Vimentin fibers, we labeled the ends of the fibers extending farthest, which could be traced continuously extending outwards. The sizes of the areas shaped by the labeled ends (the dots shown in Fig. 5A–C) and the ventricle were compared in W, M and N. The results indicated that the areas increased significantly only in W and the inside part of M (*P *< 0.05), but not the inside part of N, after the injections of *m-Emx2* Lv, in contrast to *c-Emx2* Lv or Cv (Fig. 5C, one-way ANOVA). There were no significant increases in groups injected with *m-Emx2* Lv, in contrast to those injected with Cv (Fig. 5E, *P* > 0.05, one-way ANOVA).

**Figure 7:**
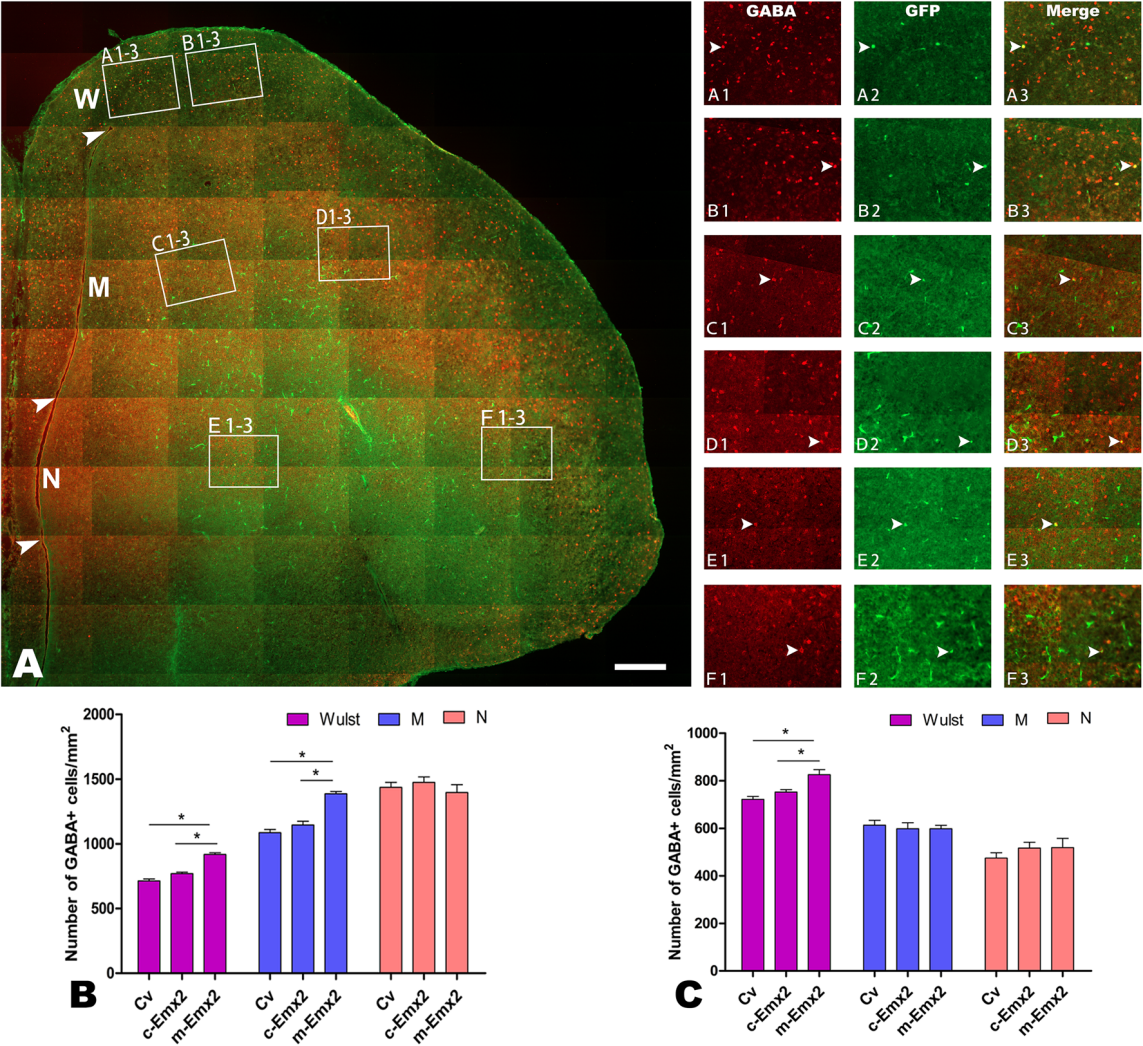
GABA distribution in the pallium at embryonic day (E) 16 after injection of lentiviruses expressing chick *Emx2* (*c-Emx2*, **B**) into the ventricle of the telencephalon at E3. The boundaries of Wulst (W), Mesopallium (M) and Nidopallium (N) are indicated by arrowheads. **A**–**F3**: GABA expression in medial or inside (**A1–3**, **C1–3** and **E1–3**) and lateral or outside (**B1–3**, **D1–3** and **F1–3**) areas is amplified in A1-F3, respectively. Some cells labeled for GABA (red) and infected with lentiviruses (expressing GFP) are indicated by arrowheads. (**B** and **C**) Comparison of the numbers of GABA cells per mm^2^ in the medial (**B**) and lateral (**C**) regions of W, M and N among the groups after injections of control lentiviruses (Cv) and *m-Emx2* or *c-Emx2* lentiviruses. Scale bar = 200 µm. The data are presented as the mean ± a standard error (SEM). *p < 0.05 (SPSS, one-way ANOVA).

For GABA or MAP2 cells, there was no obvious aggregation or lamination found in W, M and N. The densities of GABA cells in W and in the inside part of M increased significantly in the group injected with *m-Emx2* Lv compared to the groups injected with *c-Emx2* Lv or Cv (Figs. 6 and 7, *P *< 0.05, one-way ANOVA). No significant differences were found in the outside regions of these areas for GABA cells or in any of the examined areas for MAP2 among the groups injected with *m-Emx2* Lv*, c-Emx2* Lv or Cv (*P *> 0.05, one-way ANOVA). Some groups are shown in Figs. S18 and S19.

#### Gradient of neurogenesis and migration of calbindin (CB)

Given that neurogenesis gradient mainly follows an inside-out pattern in two classes of mammalian cortical neurons, projection neurons and interneurons, including most GABAergic subtypes^36–39^, we then examined the neurogenesis pattern of CB (a representative subtype of interneurons^40^), during development of W, M and N after the injections of *m-Emx2* Lv*, c-Emx2* Lv or Cv into the ventricle of the telencephalon at E3. To study the migration pattern, cells double-labeled for BrdU and CB were examined in the medial and lateral areas of W, M and N in chicks whose embryos received BrdU injection on several consecutive embryonic days (E4–E10). Cells double-labeled for BrdU and CB mainly appeared in the groups injected with BrdU at E4 to E8, and the largest percentage varied between the medial and lateral sampled areas, indicating a gradient of neurogenesis (Figs. S20–S23). We compared the largest percentages of double-labeled cells for BrdU and CB in the medial and lateral sampled areas of W, M or N among the groups receiving injections of *m-Emx2* Lv*, c-Emx2* Lv or Cv at E3. In the W and M, the largest percentages of double-labeled cells for BrdU and CB appeared earlier in the medial area, in contrast to those in the lateral area, suggesting an inside-out gradient of neurogenesis and migration in the group receiving injections of *m-Emx2* (Figs. S20 and S21), whereas an outside-in gradient of neurogenesis and migration was found after injections of *c-Emx2* or Cv lentiviruses into the ventricle of the telencephalon at E3 (Figs. S22 and S23). In the N, neurogenesis and migration followed an outside-in gradient in all the studied groups (Figs. S20–S23).

#### Anterior commissure (AC)

It has been reported that mutant mice for Emx1/2 have developmental defects in the corpus callosum, indicating that neural connections are abnormal between the two brain hemispheres^15^. As the corpus callosum is lacked in birds, we then examined whether AC (which also carries neural fibers connecting some regions in two brain hemispheres^41^) was normally formed in the studied groups at P9. Around the brain level of A8.4, AC was found in sections stained for neurofilaments expressed in neural fibers (Fig. S24A–C). AC did not show significantly abnormal features among the three groups receiving injections of *m-Emx2* Lv*, c-Emx2* Lv or Cv (n = 4 for each group)**.**

#### Behavioral assessment

As shown above, the expression levels of Reelin, Vimentin, and GABA and the gradients of neurogenesis and migration of CB changed in M after injections of *m-Emx2* Lv into the ventricle of the telencephalon at E3. It is particularly reported that the medial intermediate medial mesopallium in M is a site of memory formation in chicks^42^; thus, we examined whether the above changes in the brain pallium affect short- and long-term memory by performing passive avoidance tasks and affecting locomotor activity using the open-field test. The passive avoidance ratio, which was equal to background numbers (BN)/(BN + pecking numbers) tested at 5 min after training was higher, but not significantly, in the group receiving *m-Emx2* Lv injection in contrast to those in the groups receiving *c-Emx2* Lv or Cv injection (*P *> 0.05). However, the passive avoidance ratio tested at 120 min after training increased significantly after *m-Emx2* Lv injection compared to that after *c-Emx2 Lv* or *Cv* injection (Fig. S25A and C, *P *< 0.05, one-way ANOVA).

To assess their locomotor activities, and curious or exploratory behaviors, open-field tests were then performed for the chicks on three successive days (from post-hatching day 5 to 7). The total distance run by the animals, locomotor activity (number of squares crossed), and the percentage of time spent in the central rectangle were examined and compared among the studied groups. No obvious differences among the groups receiving *m-Emx2* Lv*, c-Emx2* Lv or Cv injection were found for the total distance of movement, the percentage of time located in the central area and the number of entries into the central area on three successive days (Fig. S26A–C, *P *> 0.05, one-way ANOVA; S Movies S1, S2, and S3).

## Discussion

### Genes involved in brain development and evolution of the mammalian pallium

Like invertebrates, the ascidian retains one *Emx* gene, while there are two in the cephalochordate amphioxus, jawless cyclostomes and gnathostomes. The two *Emx* genes, i.e., *EmxA* and *EmxB*, in the cyclostome are orthologs of *Emx1* and *Emx2* in jawed vertebrates, respectively, and *Emx3* in some fish, amphibian and marsupial species is a paralog of *Emx1*^43^. As *Emx3* is not widely distributed in vertebrates, it was not noted in the present study. In contrast to *Emx* genes, the ascidian has two copies of *Dlx* and *Nkx2.1*. These results suggest that the duplication of *Dlx* and *Nkx2.1* occurred before the splitting of subphylum urochordata from the cephalochordate and vertebrate lineages (but *Emx1/2* duplication after it). This is consistent with other reported homeobox genes involved in the development of the forebrain and midbrain (*Otx*), midbrain and anterior hindbrain (*Engrailed-1, En-1*) and axial patterning (*Hox*), as well as the midbrain–hindbrain boundary organizer (*Pax2/5/8, Pax3/7a*)^44–46^, which have a single copy or cluster in amphioxus, but two or more genes or clusters in all vertebrates^47–49^. Thus, although the above genes all support the hypothesis that one or two rounds (denoted as “1R” and “2R”) of gene duplication occurred, gene duplication started at different times during evolution. In addition, our phylogenomic analysis for reptiles is consistent with a previous study based on genomic data showing several placements of reptiles in the phylogeny of vertebrates^50^.

It is reported that the duplicated genes acquire new functions in two ways: by the evolution of new regulatory elements (allowing or suppressing gene expression in new domains) or by changes in protein sequences^9^, which is supported by the expression domains and mutant phenotypes from *Emx1/2*, *Otx1/2* and *En1/2* described above^44,45^. Given that genome-wide comparisons indicate that the human genome only has approximately 30–40% and 25% more genes than the genomes of Drosophila/Caenorhabditis and amphioxus, respectively, gene loss must occur after whole-genome duplications; however, gene loss did not occur evenly, and the genes involved in development, signal transduction and neuronal activities were preferentially retained^9,48^.

Our phylogenetic comparison of the coding sequences of the studied genes indicated that an additional fragment of ~ 20 amino acids in length was acquired in Emx1, and an additional tract of homopolymeric (Ala)_6–8_ was added to Emx2 in all the examined mammalian species except monotremes and marsupials, which have only poly-(Ala)_2_. According to previous reports, Emx1 mutant mice are viable and exhibit subtle brain defects, such as disorganized fasciculation in the anterior commissure^13^. In contrast, *Emx2* mutant or *Emx1/Emx2* double mutants exhibit many more abnormalities, including significantly reduced cerebral hemisphere size, abnormal cortical lamination, and malformed junctions between the cerebral hemispheres^14–16^. Interestingly, some phenotypes of *Emx1* and *Emx2* mutants display striking similarities to the brains of monotremes or marsupials and other nonmammals, including poor layer formation in the dorsal pallium, a lack of the corpus callosum, and some thalamocortical axons that project to the striatum^15,17,51,52^. To our knowledge, no attempts have been reported to address the extant organizations of vertebrate brains, especially their differences between several large lineages of vertebrates, from the evolutionary perspective of homeobox genes. In addition, the above data are also consistent with a reported schizencephaly with frontal lobe agenesis and absence of the corpus callosum caused by a mutation affecting the splicing of the first intron of *Emx2*^53^. These reports will deepen our understanding of homeobox gene changes in vertebrate evolution and human neuropathy.

It is needed to point out that a subset of radial glial progenitors which generates only upper-layer neurons and contributes to the development of callosal neurons in the developing mammalian brain is absent from avian brain lineage of selected Emx2 +, and there are no homologous delayed radial glial progenitors in any sectors of the chick forebrain. These are regraded to be possibly associated with the evolution of the corpus callosum^54,55^. According to our study, the absence of the corpus callosum in non-Eutherian species is probably related to the differences in the coding sequences of Emx1 and Emx2.

### Additional tract of homopolymeric amino acids and possible functions

Similar to Emx1/2, poly-(Ala)_6_ only appears in En1, but not in its paralog, En2, and poly-(Ala)_6_ is the minimal domain for the transcriptional activity of En1^13,15,17,56^. In addition, it has been reported that at least 17 human hereditary neurological diseases are caused by the excessive expansion of homopolymeric amino acids with repeat lengths ranging from 7 to 14, of which 16% are poly-Ala^43^. However, the proportions of amino acids containing homopolymeric amino acid tracts of glutamine (Q), A (alanine), G glycine (G) and proline (P) were no less than 0.5% after analyzing three protein datasets from human, zebrafish and sea lamprey^43^. Such expansion generally, but not always, arises through replication slippage or unequal recombination and is dominantly associated with transcriptional regulation and some neural diseases, such as Huntington’s disease and mental retardation^57,58^.

Our study further indicated that, compared to *Emx1* and *Emx2, Dlx2* or *Nkx2.1* were more conserved during evolution, which is reflected in their coding sequences, and started gene duplication before the split of urochordates from the cephalochordate and vertebrate lineages, although they undertook the same Darwin selection, and all share a relatively conserved gene synteny^59^. Thus, these data indicate that the genes involved in the development of the pallium or subpallium underwent different changes during evolution. This partly answers the question of why the mammalian telencephalon is a mosaic structure.

A previous report indicated that neurogenesis in W partly follows the inside-out pattern, as in mammalian dorsal cortex^60^, but it follows the outside-in pattern in M and N^61^, which are homologous to mammalian lateral and ventral pallium, respectively^11,29–31^. Like projection neurons in the mammalian cerebral cortex, most interneurons follow an inside-out pattern of neurogenesis^36–39^. Here we examined the pattern of neurogenesis in a representative subtype of GABAergic neuron. Our results showed that the neurogenesis pattern of CB neurons in W and M, but not in N, changed towards the one which appears in mammalian cortex^40^ after infection with *m-Emx2* Lv but remained unchanged significantly after infection with *c-Emx2* Lv or Cv. This is consistent with the results indicating that Reelin, Vimentin and GABA changed in W and in the inside part of M but not in N. The above result also agrees with the finding that the overexpression of Reelin caused more regularly distributed radial glia with longer fibers, and cells expressing Reelin and Vimentin are concerned with cell migration^33^. It is noted that *Emx1* is not expressed in N^11,12^. However, it is unknown whether the changes in the phenotypes reported in *Emx2* mutants were not observed in N is related to the lack of Emx1 expression in N (or the lack of synergistic effects of Emx1 and Emx2^13–17^).

In addition, our results indicated that the above changes did not appear in the whole M, which might be because we only generated a chimeric brain, and only some cells were infected with lentiviruses expressing target genes. Thus, brain development was only affected partly, and this probably also caused no significant differences in locomotor activity tested in the open field.

It is generally accepted that, on the basis of histochemical, gene expression and hodological data, the pallium is regionalized into roughly comparable medial, dorsal and lateral pallial regions, of which the lateral pallium is Emx-1-positive, including M in birds, and the dorsolateral claustrum in mammals, while the ventral pallium is Emx-1-negative, including N in birds, and the ventral part of the piriform cortex, the ventromedial claustrum and the lateral and basomedial amygdalar nuclei^11,28–30,62^. The reports from Emx1, Emx2 or Emx1/2 double mutants indicate that Emx1 and Emx 2 have separate and synergistic effects on the organization of medial, dorsal and lateral pallium^13–17^ and our present study has shown that the genesis pattern of CB neurons in N (the ventral pallium lacking Emx1 expression) did not change after infection with m-Emx2 or c-Emx2 Lv. However, it needs to be further studied whether some changes in organization between the ventral pallium and the other three parts of pallium is due to the variance in the expression of Emx1.

The present results indicated that the passive avoidance ratio increased significantly in the group infected with *m-Emx2* Lv compared with the groups infected with *c-Emx2* Lv or Cv, which might be related to the increase in the GABA expression in the medial part of M, which is involved in chick memory^41^. As mentioned above, M is homologous to the mammalian lateral pallium, and our present results are in agreement with previous reports in mammals to indicate that some regions in the lateral pallium are involved in cognitive processes (such as learning and memory)^63–65^.

In addition, there were no significant differences between the chicks infected with *c-Emx2* Lv or Cv, this might be explained by a scenario: *c-Emx2* amount produced in the chick brain is enough for its actions in the brain development. Our study indicated that variations in Emx2 might be involved in the divergent organization and evolution of the pallium, such as neuron positioning during migration and the absence of the corpus callosum in monotremes and marsupials. However, it could not be true that a sole gene is involved in the organization and evolution of the mammalian pallium, and other genes must be also involved. Further studies are needed to explore what genes are involved and how they are performed.

### First delivery of viral vectors for target gene expression into the avian brain

Chicken embryos are widely used in developmental biology due to the ready availability of fertile eggs, their well-documented stages of embryology, and their early sequenced genomes^60,66^. However, it is difficult to isolate a single pronucleus on a large yolky oocyte, resulting in few transgenic birds^67,68^. Although this is partly overcome by using viral vectors to deliver target genes directly into early embryos, yielding somatic chimeras or germline transgenesis^69,70^, no reports have been concerned with the brain. As shown above, the whole telencephalon was homogeneously infected after pLVX-mCMV-ZsGreen lentiviruses were injected into the ventricle of the telencephalon at E3, and the embryos could develop to hatching normally (the hatching rate was above 85%). Our study is approximately consistent with the two previous reports in which a large number of cells were successfully infected with viruses containing GFP after they were injected at E0 into the subgerminal cavity below the embryo, and the injected embryos also survived to hatching^69,70^. Thus, we first provide a method for engineering chimeric brains expressing target genes at high levels, opening a new avenue for the genetic manipulation of avian brains.

## Materials and methods

Fertilized eggs (embryonic day 0) and KM (Kunming) mice which are the descendants of Swiss from Switzerland were obtained from Beijing Vital River Laboratory Animal Technology Co., Ltd. All experiment procedures were reviewed and approved by the Animal Management Committee of the College of Life Sciences, Beijing Normal University. The mice and chicks were cared for according to the guidelines set forth by the Beijing Laboratory Animal Welfare and Ethics Review guidelines. The experimental protocols and methods in this study were carried out in compliance with the ARRIVE guidelines.

Given that our primary goal was to determine whether gene variations are related to the novelty of the mammalian dorsal pallium, the genes examined in the present report were previously well studied and shown to be exclusively involved in the development of the subregions of the telencephalon^10,11^. The following homeobox genes were studied: *Emx1/2, Pax6, Dlx2* and *Nkx2.1.* The full-length proteins or mRNA sequences of these genes from approximately 500 species whose names are shown in Fig. S1–S5 were downloaded from NCBI (http://www.ncbi.nlm.nih.gov/) or Ensembl (https://asia.ensembl.org/index.html), which were renewed until the end of April 2022. Species whose complete genomes have been sequenced and annotated were used to perform genomic analysis of gene duplication/loss at the whole-genome level. The analyses covered all of the major phylogenetic lineages of chordates, including urochordata and cephalochordata (Table S1).

The protein sequences encoded by the examined full-length genes were fully aligned using ClustalW set at the default parameters^71^ and trimmed with trimAl v1.2 with no gap allowed and a similarity threshold of 0.0005^72^. However, those sequences were not aligned and further analyzed, as some amino acids (even highly conserved) were largely different from those of other examined species. Neighbor-joining phylogenetic trees were then generated (Dayhoff Model) and displayed using MEGA11 with 1000 bootstraps^73^.

### Synteny analysis and calculation of Ka/Ks values

For synteny analysis, the locations of the studied genes and 30 neighboring genes were compared by using Ensembl (https://asia.ensembl.org/index.html) or NCBI (https://www.ncbi.nlm.nih.gov/). The chromosome localization maps of these genes were drawn by MapChart 2.32^74^. To calculate nonsynonymous (Ka) and synonymous (Ks) substitutions per site in each protein-coding region of the studied genes, we used KaKs_Calculator 2.0^75^ under the LPB model. Fisher’s exact test was used to determine adaptive selection, and the z test (Condon-Based Z Test for Selection, 1000 cycles) was used to calculate the deviation of the Ka/Ks ratios from neutrality (Ka/Ks = 1)^76^.

### Tertiary structure prediction

Tertiary structure prediction was performed using the Rosetta server^77^ (http://robetta.bakerlab.org/). Secondary structure and domain predictions were performed using the online servers PredictProtein^78^ (http://www.predictprotein.org/), GlobPlot2.3^79^ (http://globplot.embl.de/), and Dompred^80^ (http://bioinf.cs.ucl.ac.uk/dompred). The structure was then analyzed using PyMOL.

### Construction of the expression vector pcDNA3.1

All of the coding regions of mouse and chicken Emx1 and Emx2 and anole Emx2 were amplified via PCR from mouse or chicken embryonic brain cDNA (E13.5 and E8 for mouse and chicken, respectively). The entire cDNA of the shortened poly-(Ala)_7_ to (Ala)_5_ of mouse *Emx2* was synthesized*.* The amplified or synthesized DNA fragments were then cloned into the expression vector pcDNA3.1 (Invitrogen). Proper orientation and reading frames were verified by sequencing analysis.

### Primary neuronal cultures and transfection with the expression vector pcDNA3.1

Chick embryos (E8) were collected and decapitated. The brains were removed under a binocular microscope and maintained in cold HBSS supplemented with 0.6% glucose without Ca^2+^ or Mg^2+^ [calcium and magnesium-free (CMF)-HBSS-G]. The cerebral hemispheres were isolated, triturated, and incubated in CMF-HBSS-G containing 0.125% trypsin (Invitrogen) in 1 ml of DMEM/F12 (Invitrogen) for 5 min at 37 °C. The suspension was then washed with DMEM/F12, and the cells were seeded onto six-well plates coated with a cell attachment matrix (poly-L-lysine; Sigma) at a density of 1 × 10^6^ cells per well in DMEM/F12 supplemented with 10% FBS and penicillin–streptomycin (Invitrogen).

After culturing for 24 h, these cells were cotransfected with the expression vectors and pEGFP-N1 at a ratio of 3:1 (μg) using ExGen 500 in vitro transfection reagent (Fermentas). The pEGFP-N1 plasmid expresses cytoplasmic EGFP (enhanced green fluorescent protein) as an independent marker of successful transfection. After incubation for 48 h, when EGFP was highly expressed, the cultures were homogenized for RNA and protein extraction.

### RNA extraction and qRT–PCR

RNA was extracted from cultured cells or E8 brains using TRIzol (Invitrogen), and cDNA was generated using MLV reverse transcriptase (TIANGEN, China) and amplified using gene-specific primers. Quantitative RT–PCR was then performed using a Quantitect SYBR Green PCR master mix (TransGen, China) with a 7900HT Sequence Detection System (Applied Biosystems). Each reaction was standardized to a GAPDH control.

### Protein extraction and western blot analysis

The cultured cells were lysed in 50 mM Tris (pH 7.4), 150 mM NaCl, 1% Triton X-100, and 0.1 mM phenyl methane sulfonyl fluoride. The samples were separated via 6% SDS–PAGE for Reelin or 12% for GAPDH and were electrotransferred to a polyvinylidene-difluoride membrane (Millipore). The membranes were incubated in either a Reelin-specific monoclonal antibody (clone 142, Chemicon) or anti-GAPDH (Santa Cruz) overnight at 4 °C in PBS-Tween 20. After incubation in a secondary antibody conjugated to HRP (goat anti-mouse antibody for Reelin or goat anti-rabbit antibody for GAPDH, Santa Cruz), chemiluminescence detection was performed using ECL (Millipore). The volume density of each Reelin band was quantified using ImageJ software and was normalized to that of the corresponding GAPDH band.

### Construction and injections of lentivirus-Emx2 vectors

Mouse and chick *Emx2* were synthesized according to the sequences in NCBI (mouse: XM_004177159.1; chick: KT932702). They were inserted into a vector (pLVX-mCMV-ZsGreen, Shen Zhen BaiEnWei) to generate pLVX-*m-Emx2*-mCMV-ZsGreen (*m-Emx2* Lv*)* or pLVX-*c- Emx2*-mCMV-ZsGreen *(c-Emx2* Lv*)*, respectively. The titers were 6–8 × 10^8^ TU/ml.

After 3 d of incubation of fertilized eggs in the incubator (99.5 °F, 60–70% relative humidity), the eggs were removed and sterilized using 70% alcohol. To gain access to the embryo, a 4-by-4-mm window was drilled at the top of the eggshell with a handheld rotary tool, and the shell membrane was removed with forceps. Under a stereoscopic microscope (Scoptic, China), the vesicle of the telencephalon was located over the eye and the beating heart, the two of which were easily seen. A glass electrode with a diameter of 25 µm was inserted into the telencephalon vesicle, and 3 µl of lentivirus liquid (2.0 × 10^9^ TU/ml) containing *m-Emx2* Lv*, c-Emx2* Lv *or* control virus (Cv, pLVX-mCMV-ZsGreen with no targeted gene inserted), mixed in 20:1 with 0.1% Fast green (Sigma), was injected over 15–25 min. The injection was successful if the viral solution in green spread only in the vesicle of the telencephalon.

After injection, the hole was sealed with a piece of clean parafilm and medical adhesive tape to prevent microbial contamination and fluid loss during incubation. The eggs were placed blunt end up, returned to the incubator, and turned periodically. Embryonic development continued until either E12, E16 or the natural end of hatching. The eggs hatched out after 20 days of incubation and were subsequently kept in a heated brooder. More than 75% of fertilized eggs treated with the above operation successfully hatched out.

### Nissl staining and immunohistochemistry

After the chicks were deeply anesthetized with sodium pentobarbital and then sacrificed with a perfusion of ice-cold saline through the heart, followed by 4% cold paraformaldehyde in 0.1 M phosphate-buffered saline (pH 7.4). The brains were postfixed for 6 h in the same fixative at 4 °C. E12 or E16 brains were directly placed into the post fixative without perfusion. After fixation, the brains were immersed in 30% sucrose at 4 °C overnight. They were embedded in Jung compound (Leica) and cut into 10 μm thick slices with a freezing microtome (Leica). Every fifth section was mounted onto a poly-L-lysine-coated slide. A total of six sets of sections were collected from each brain and processed for Nissl staining or immunohistochemistry.

The sections were incubated in a permeabilization/blocking buffer with 5% normal horse or donkey serum that was soluble in Triton X-100/PBS) for 30 min at room temperature. They were then incubated with a primary antiserum, including mouse anti-Reelin (Santa Cruz, sc-25346, 1:300), anti-neurofilament (Chemicon, MAB1621, 1:600), anti-Vimentin (Chemicon, VM 3B4, 1:200), rat anti-BrdU antibody (AbD Serotec, 1:2000), rabbit anti-MAP2 (Chemicon, AB5622, 1:400), anti-CB (Swant, Bellinzona, Switzerland, 1:2,000) or anti-GABA (Sigma, A2052, 1:1000), in blocking buffer overnight at 4 °C. The specificity of the primary antibodies was verified in previous reports and our preliminary experiment. For BrdU immunohistochemistry, the sections were pretreated with 2 N HCl for 3 h, followed by Borax buffer (0.1 M, pH 8.4) for 30 min.

For immunofluorescent staining, the sections were incubated with goat anti-mouse IgG (H + L) conjugated to Alexa Fluor 488 (1:100, Molecular Probes), donkey anti-rat IgG (H + L) conjugated to Texas Red (Jackson; 1:400), or DyLight 488-conjugated sheep anti-rabbit IgG (1:400, Jackson ImmunoResearch, Cat. 313-486-003), corresponding to the primary antibody.

To study the migration pattern of calbindin neurons, a single dose of BrdU was injected into chick eggs on consecutive embryonic (E) days (E4–E8) when most neurons in the pallium were generated to date neuronal birthdays. According to the sizes of embryos, the different doses of BrdU were injected into an area adjacent to a large vessel of the hatching eggs on different embryonic days (1 µl for E4, 1.3 µl for E5, 2 µl for E6, 3 µl for E7, and 6.8 µl for E8 (Sigma, 50 mg/ml). At E14, the chicks were removed from the egg and deeply anesthetized, perfused and fixed. The brain sections were then processed for double immunofluorescent staining for BrdU and CB, as described above.

### Behavioral assessment

To perform the one-trial passive avoidance task, chicks at 1 d of age were placed in pairs in pens (20 × 15 × 15 cm) one hour before training. The pens were illuminated by an overhead light (25 W) and were maintained at 25–30 °C. The test of the one-trial passive avoidance task was performed according to the following processes:

1. *Pretraining* The chicks were pretrained by a single presentation of a small (2 mm) white bead coated with pure water for 10 s, and the number of times the bead was pecked was recorded. After 20 min, the above test was repeated. After 30 min, the chicks were given a single presentation of a small (3 mm in diameter) red bead coated with pure water for 10 s, and the number of times the bead was pecked was recorded during the time (10 s) as the background number (BN).
2. *Training* After 30 min of pretraining, the chicks were given a single presentation of a red bead with the same size as in the pretraining but coated with methyl anthranilate (MeA). Chicks that pecked this bead and evinced a disgust response were considered to be trained.
3. *Test* The chicks were given a single presentation of a red bead with the same size as before but coated with nothing for 10 s after 5 min (test for short-term memory) or 120 min (test for long-term memory) of training. The pecking numbers (PN) were recorded during the time (10 s). Each chick was trained and tested only once. The avoidance ratio (AR), which was defined as BN/(BN + PN), was compared among the studied groups using the χ^2^ test of independence.

Open-field tests were performed for the chicks to assess their locomotor activities, and curious or exploratory behaviors on three successive days (from post-hatching day 5 to 7). The open-field was formed with dark Formica flooring (77 × 56 × 32 cm). The tests were carried out in a room lit by a 60-W light 1.75 m above the center of the open field (150 lx at the center of the apparatus). The experimental sessions were recorded with a video camera fixed above the center of the open field by a holder standing on the ground and analyzed by Xeye Fcs (Beijing, China). Each chick at P5 was placed in the center of the open field and allowed to freely move for 15 min. After each test, the open field was cleaned with 75% ethanol and then dried with a dry cloth. To assess locomotor activity, a central rectangle was boxed from the image of the open field on the television screen, corresponding to 38 cm × 28 cm in the open field. The total distance of movement, the percentage of time spent in the central area and the number of entries into the central area were assessed on three successive days. For the above behavioral tests, each chick was tested singly, the chicks were coded ahead and the researchers were blinded to the codes until all the analyses were finished.

### Image acquisition and data statistics

The immunofluorescent images were taken with a ZEISS inverted fluorescence microscope (AxioCam MRm, Zeiss) under a 20 × or 40 × objective lens that was equipped with a monochromatic digital camera (AxioCam MRm, Zeiss). AxioVision Rel. 4.8 acquisition and processing software was used to acquire uniform digital images for each antibody throughout the experiments, and the images were converted to TIFF files. ImageJ and Adobe Photoshop were used to analyze and manage the TIFF files.

As the present study was mainly concerned with whether brain changes appeared among the W, M, N and St, the brain levels (from A6.2-A10.2 in the chick brain atlas) containing the above regions were chosen. There were no significant differences among these brain levels, and the results reported were combined.

The obtained values (the numbers of labeled cells) were compared by using the SPSS 11.5 software package. One-way ANOVA was conducted to compare the differences among the studied groups. Before ANOVA, the distributions of dependent variables were tested for normality, and homogeneity of variance was assessed for equality of error variance (Levene’s test). The data are presented as the mean ± SEM. Differences between the groups of data were considered either nonsignificant (*p *> 0.05) or significant at various levels (*p < 0.05, **p < 0.01 and ***p < 0.001).

### Supplementary Information

Supplementary Information 1.

Supplementary Movie 1.

Supplementary Movie 2.

Supplementary Movie 3.

## Data Availability

The datasets for the full-length proteins or mRNA sequences of the genes analyzed during the current study are available at https://www.ncbi.nlm.nih.gov/gene/ or at https://asia.ensembl.org/index.html.
