# Supplementary material for: Comparison of genes involved in brain development: insights into the organization and evolution of the telencephalic pallium
Source: Sci Rep. 2024 Mar 13;14:6102. doi: 10.1038/s41598-024-51964-1 (PMC10937912; doi:10.1038/s41598-024-51964-1)
Supplement: Supplementary file 1 — Supplementary Information 1. [file 41598_2024_51964_MOESM1_ESM.pdf]

Supporting Information for

Comparison of Genes Involved in Brain Development—Insights into the Organization and Evolution of the Telencephalic Pallium

Jiangyan Zhang, Rui Zhao, Shiying Lin, Dong Yang, Shan Lu，Zenan Liu, Yuanyuan Gao, Yiyun Zhang, Bing Hou, Chao Xi, Jin Liu, Jie Bing, Erli Pang, Kui Lin, Shaoju Zeng

Shaoju Zeng
Email: sjzeng@bnu.edu.cn

This PDF file includes:

Figures S1 to S26
Tables S1 to S3
Legends for Movies S1 to S3
SI References

Other supporting materials for this manuscript include the following:

Movies S1 to S3

Fig. S1.

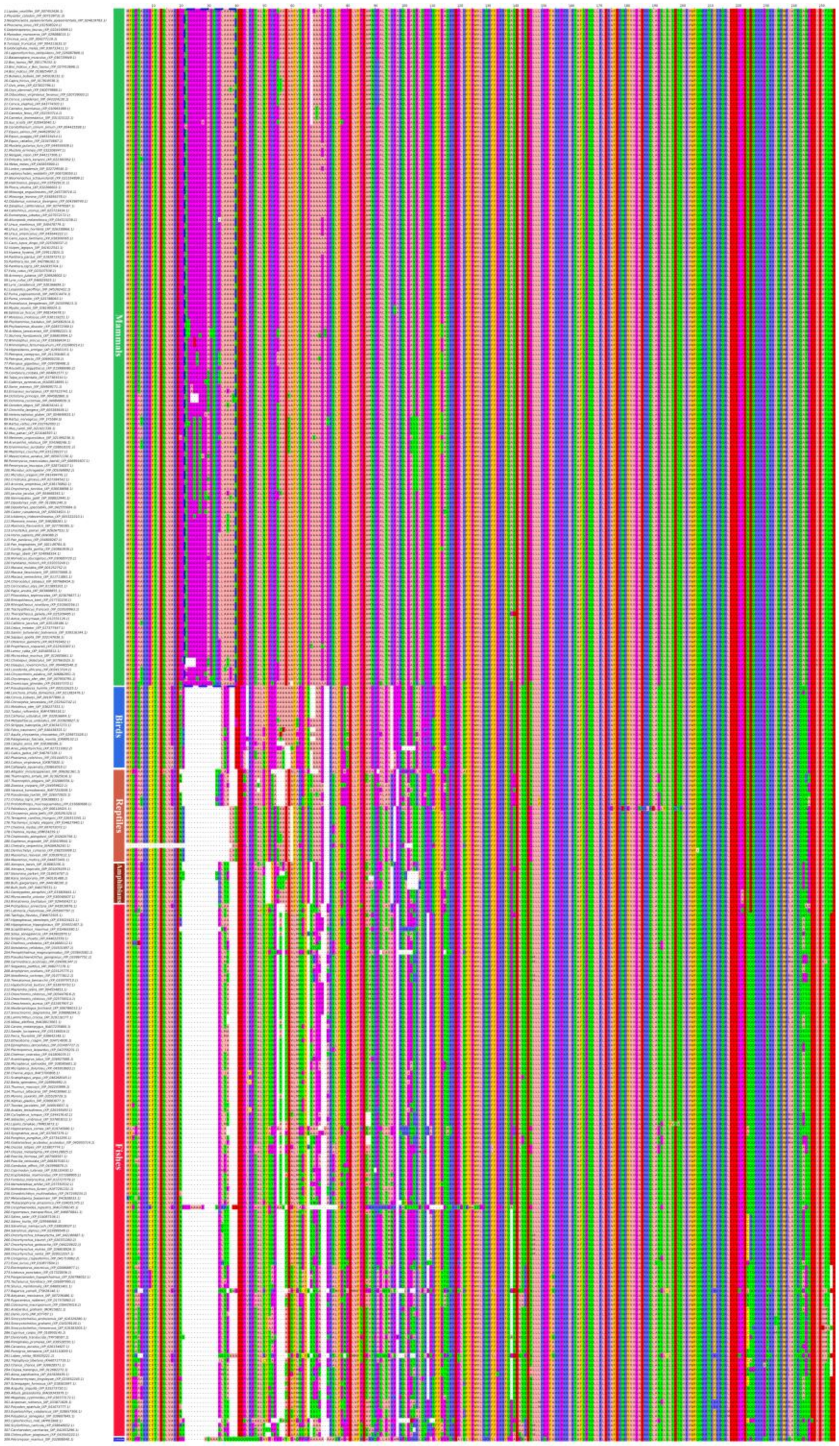

Fig. S1 Alignment of the protein sequences of Emx1 from 309 vertebrate species. An additional fragment of ~20 amino acids in length is present in all of the eutherians and marsupials (M) (boxed, indicated by an arrowhead). Cyc: cyclostome.

Fig. S2.

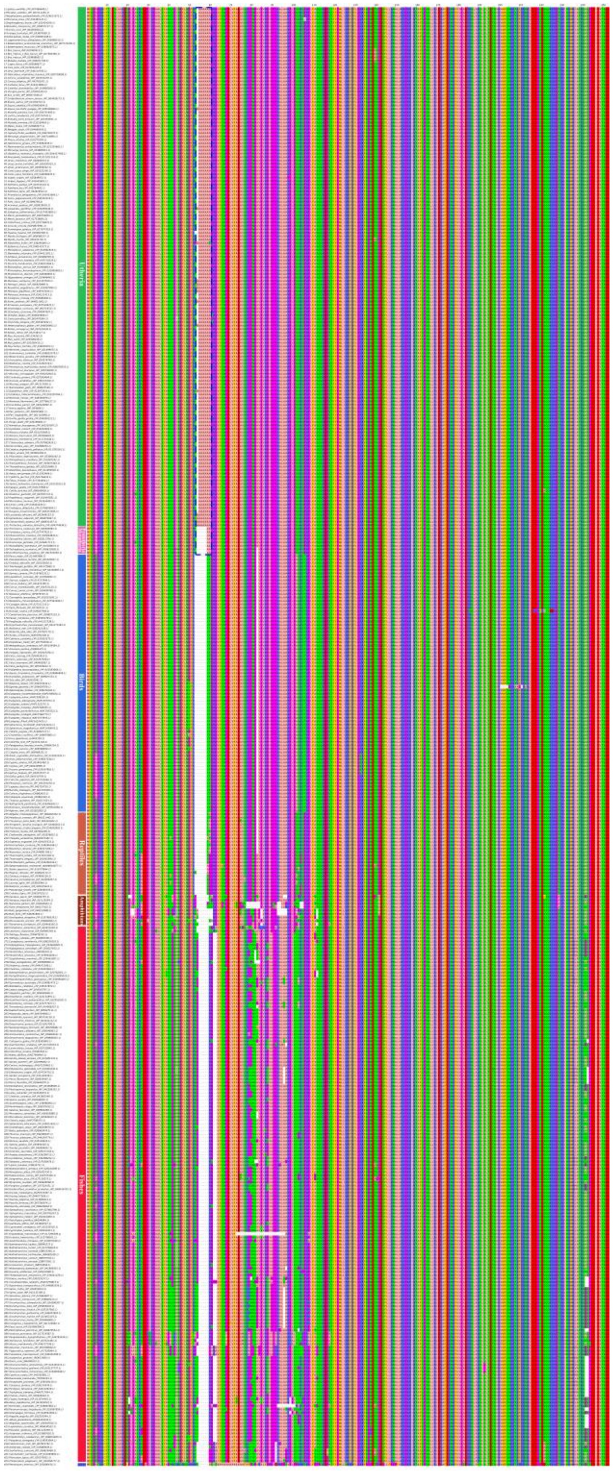

Fig. S2 Alignment of the protein sequences of Emx2 from 424 vertebrate species. There is a tract of 6-8 alanine (A) residues in all studied Eutheria and a tract of 2 A residues in all studied Prototheria and Metatheria (boxed, indicated by an arrowhead). Cyc: cyclostome.

Fig. S3.

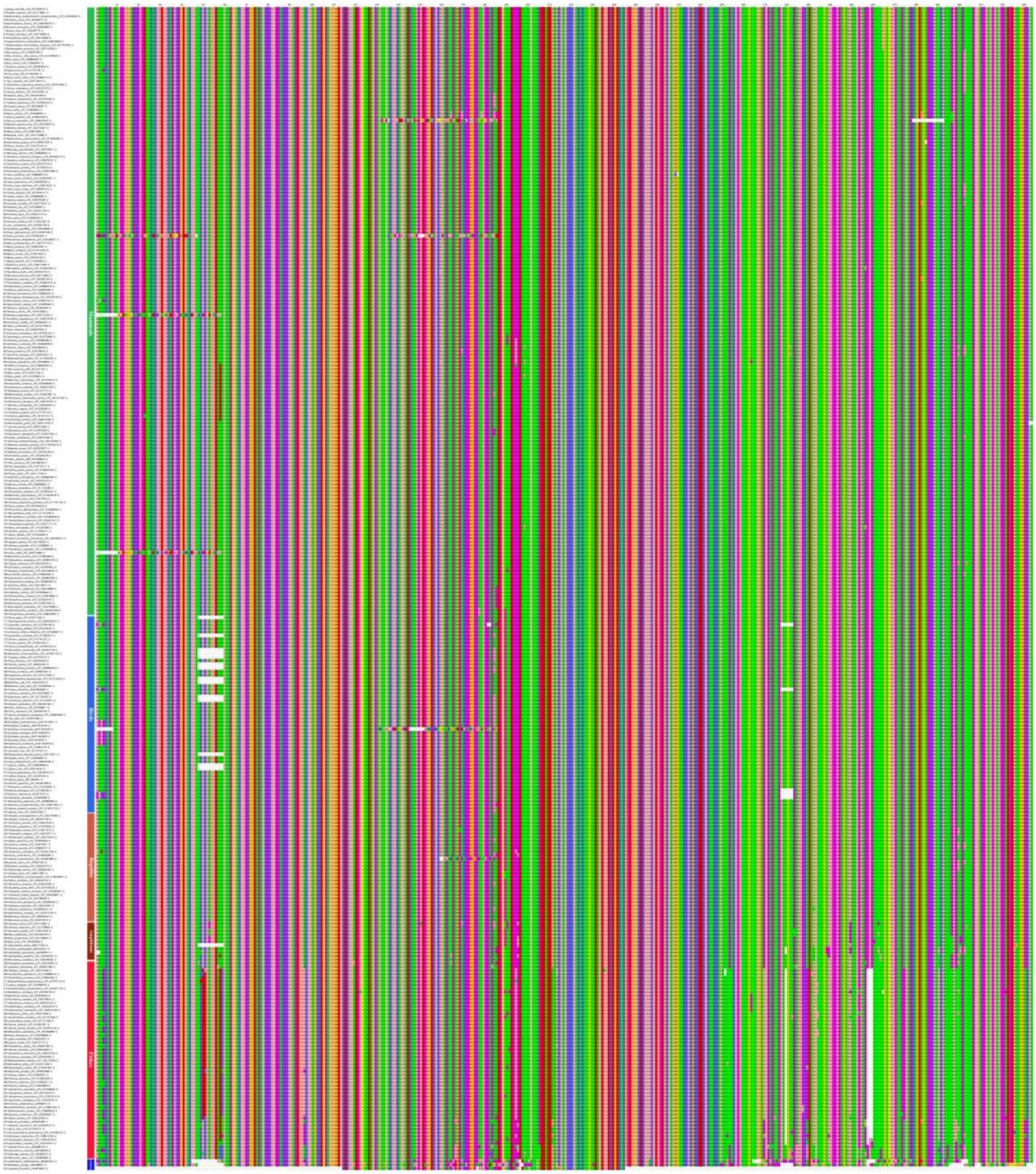

Fig. S3 Alignment of the protein sequences of Pax6 from 323 vertebrate species. Cyc: cyclostome.

Fig. S4.

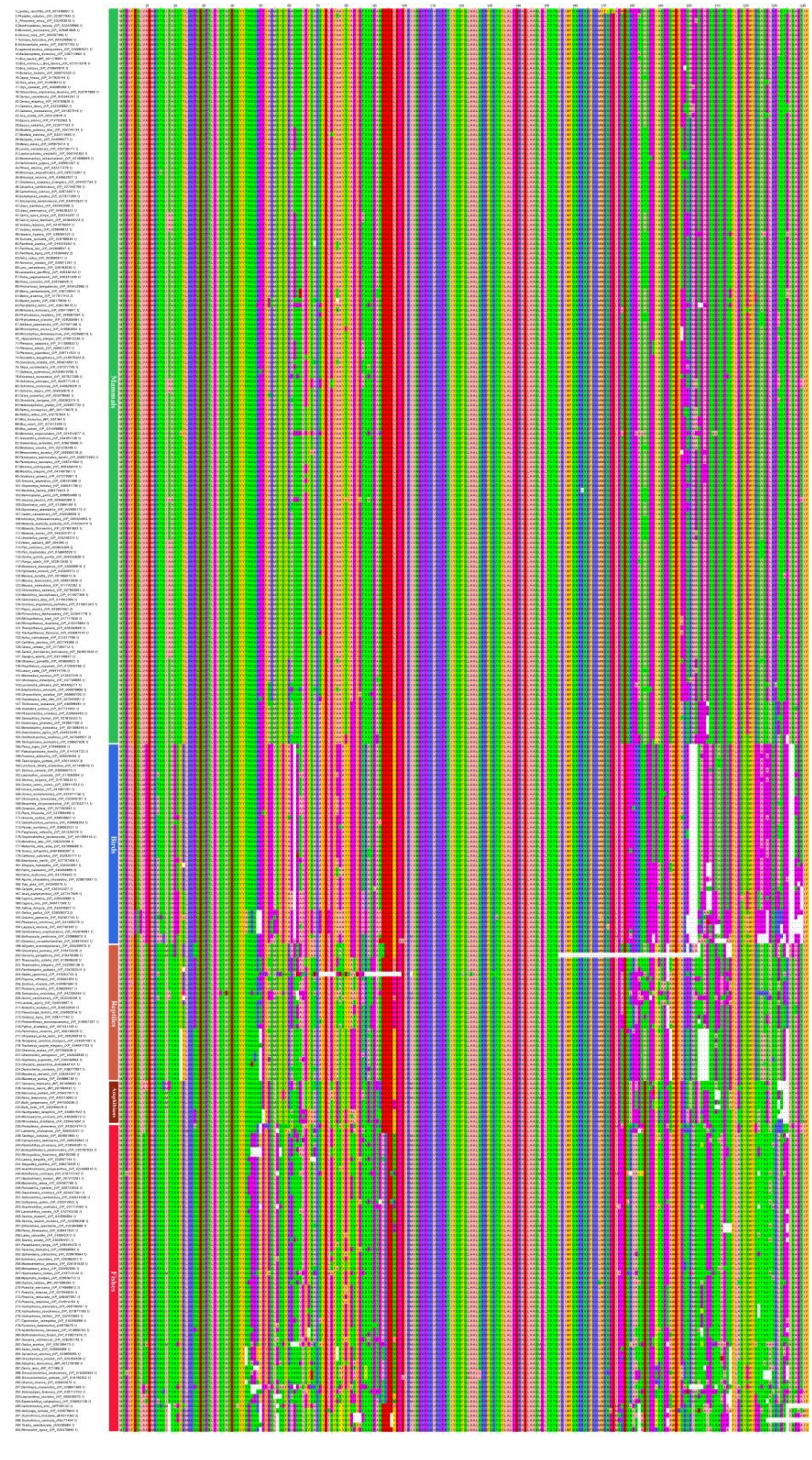

Fig. S4 Alignment of the protein sequences of Dlx2 from 300 vertebrate species. Cyc: cyclostome

Fig. S5.

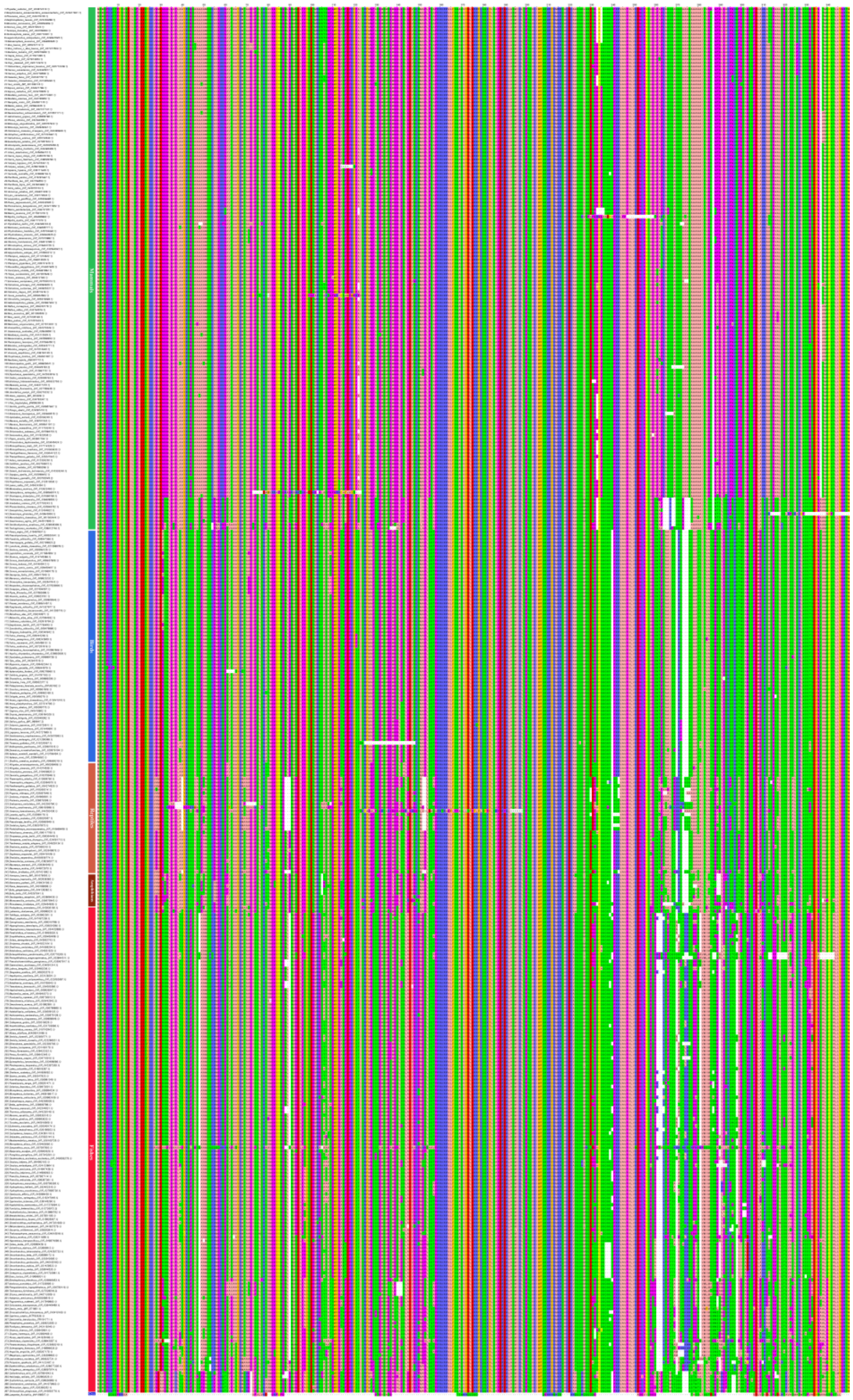

Fig. S5 Alignment of the protein sequences of Nkx2.1 from 308 vertebrate species. Cyc: cyclostome.

Fig. S6.

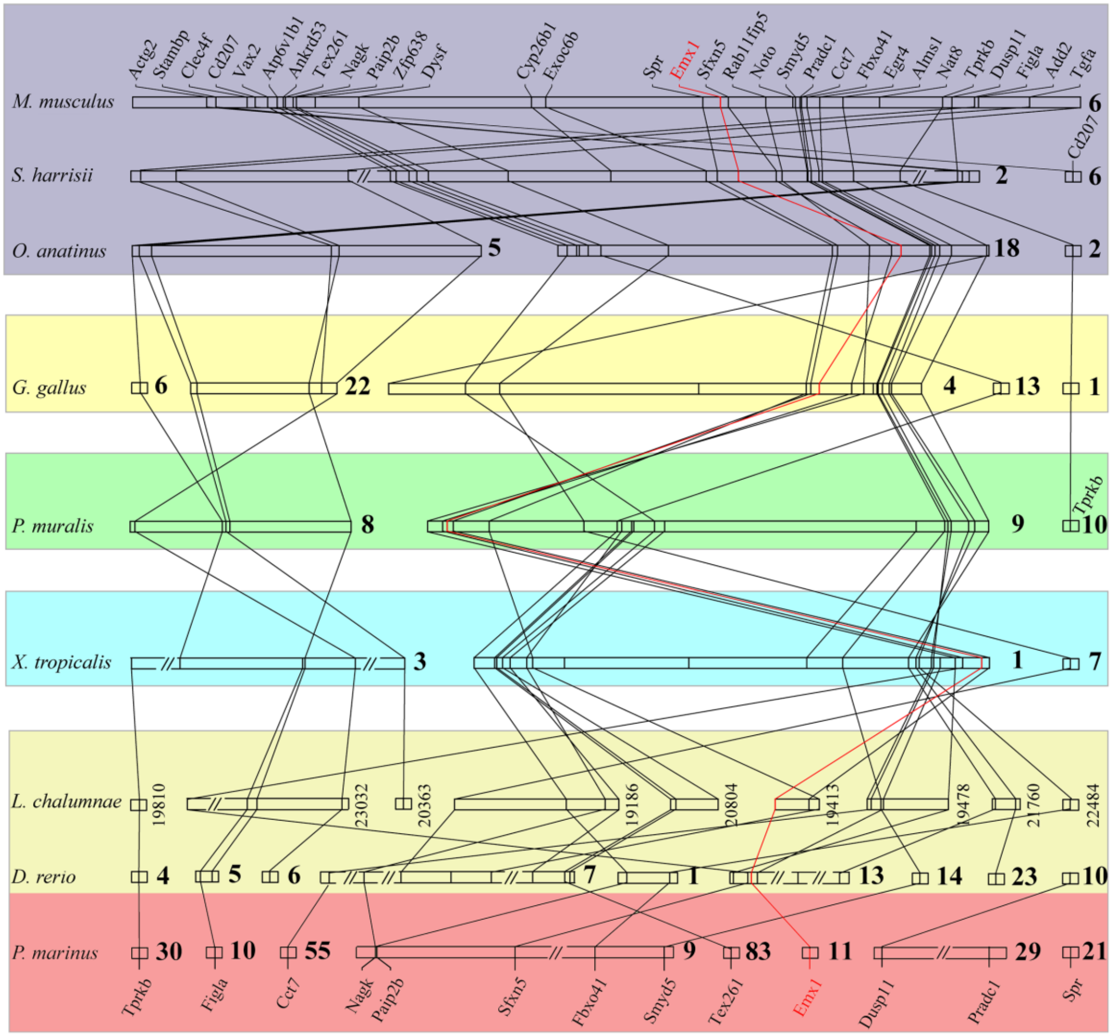

Fig. S6 Synteny of mouse *Emx1* (red) and its neighboring genes within 2.7 Mb chromosome (Chr) length. Numbers to the right of the box indicate Chr No. The compared species include monotreme (*Ornithorhynchus anatinus*), marsupial (*Sarcophilus harrisii*), chick *(Gallus gallus)*, gecko *(Podarcis muralis)*, Xenopus *(Xenopus tropicalis)*, Latimeria (*Latimeria chalumnae)*, zebrafish *(Danio rerio)* and lamprey *(Petromyzon marinus)*. The chromosome localization maps were drawn by MapChart 2.32[1].

Fig. S7.

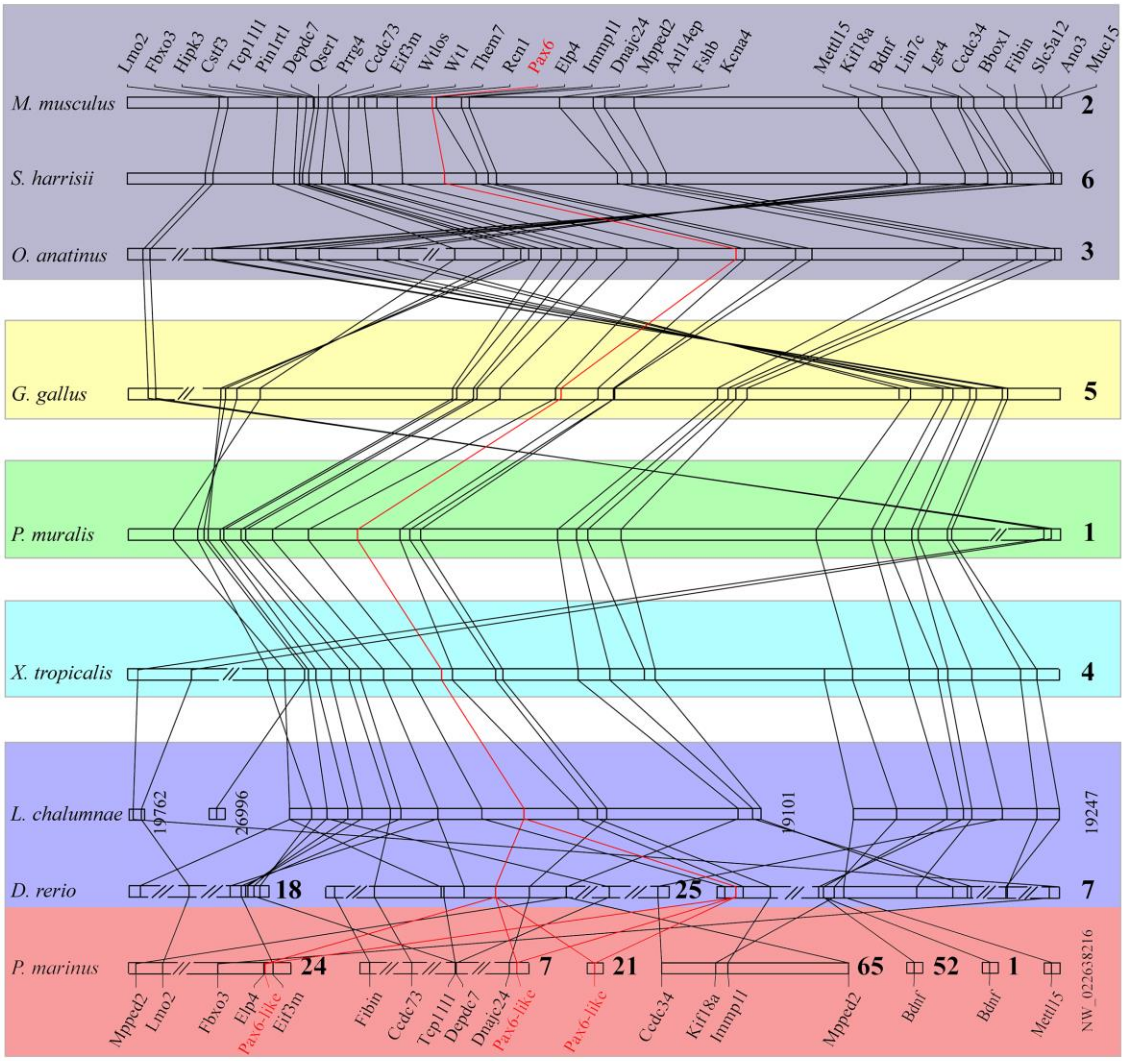

Fig. S7 Synteny of mouse *Pax6* (red) and its neighboring genes within 6.8 Mb chromosome (Chr) length. Numbers to the right of the box indicate Chr No. The compared species include monotreme (*Ornithorhynchus anatinus*), marsupial (*Sarcophilus harrisii*), chick *(Gallus gallus)*, gecko *(Podarcis muralis)*, Xenopus *(Xenopus tropicalis)*, Latimeria (*Latimeria chalumnae)*, zebrafish *(Danio rerio)* and lamprey *(Petromyzon marinus)*. The chromosome localization maps were drawn by MapChart 2.32[1].

Fig. S8.

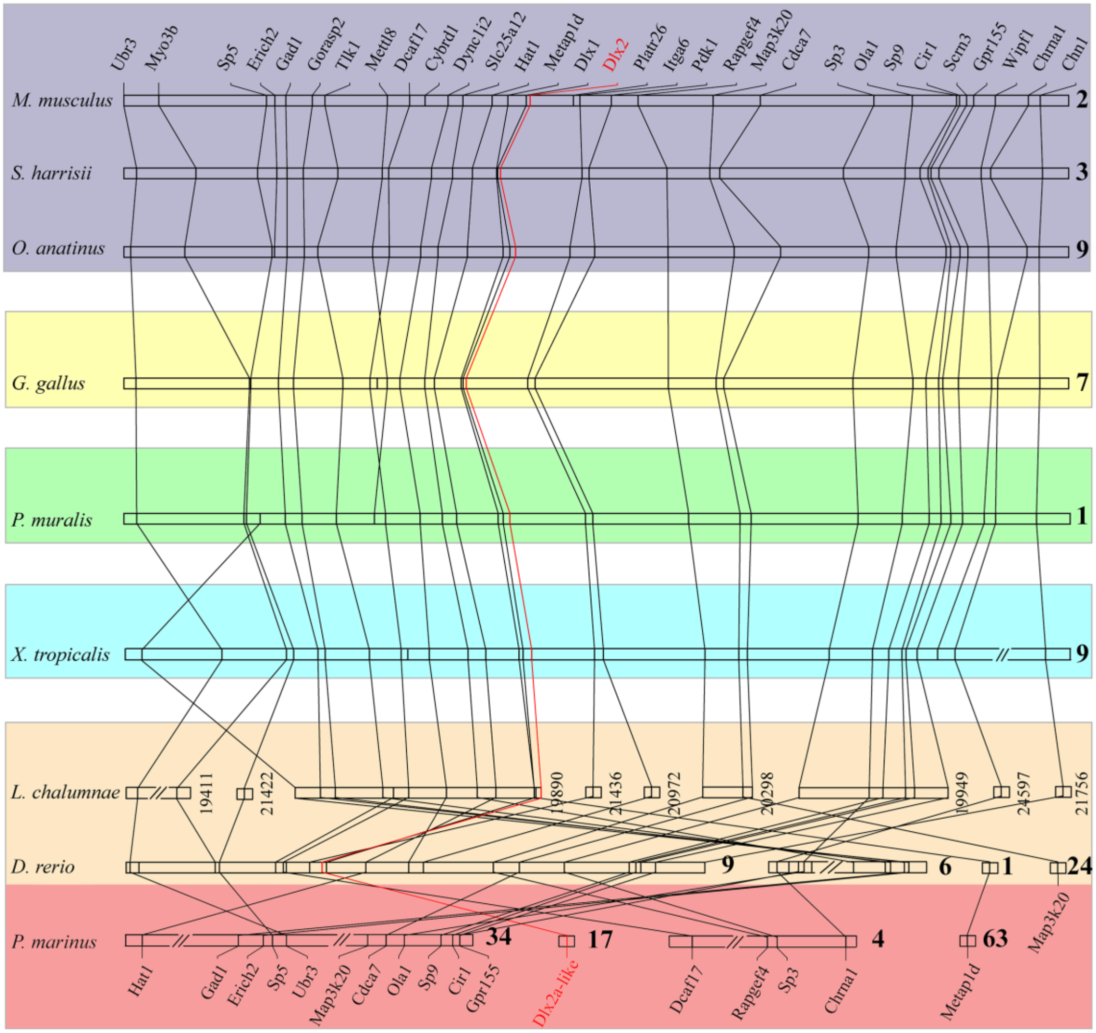

Fig. S8 Synteny of mouse *Dlx2* (red) and its neighboring genes within 3.7 Mb chromosome (Chr) length. Numbers to the right of the box indicate Chr No. The compared species include monotreme (*Ornithorhynchus anatinus*), marsupial (*Sarcophilus harrisii*), chick (*Gallus gallus*), gecko (*Podarcis muralis*), Xenopus (*Xenopus tropicalis*), Latimeria (*Latimeria chalumnae*), zebrafish (*Danio rerio*) and lamprey (*Petromyzon marinus*). The chromosome localization maps were drawn by MapChart 2.32[1].

Fig. S9.

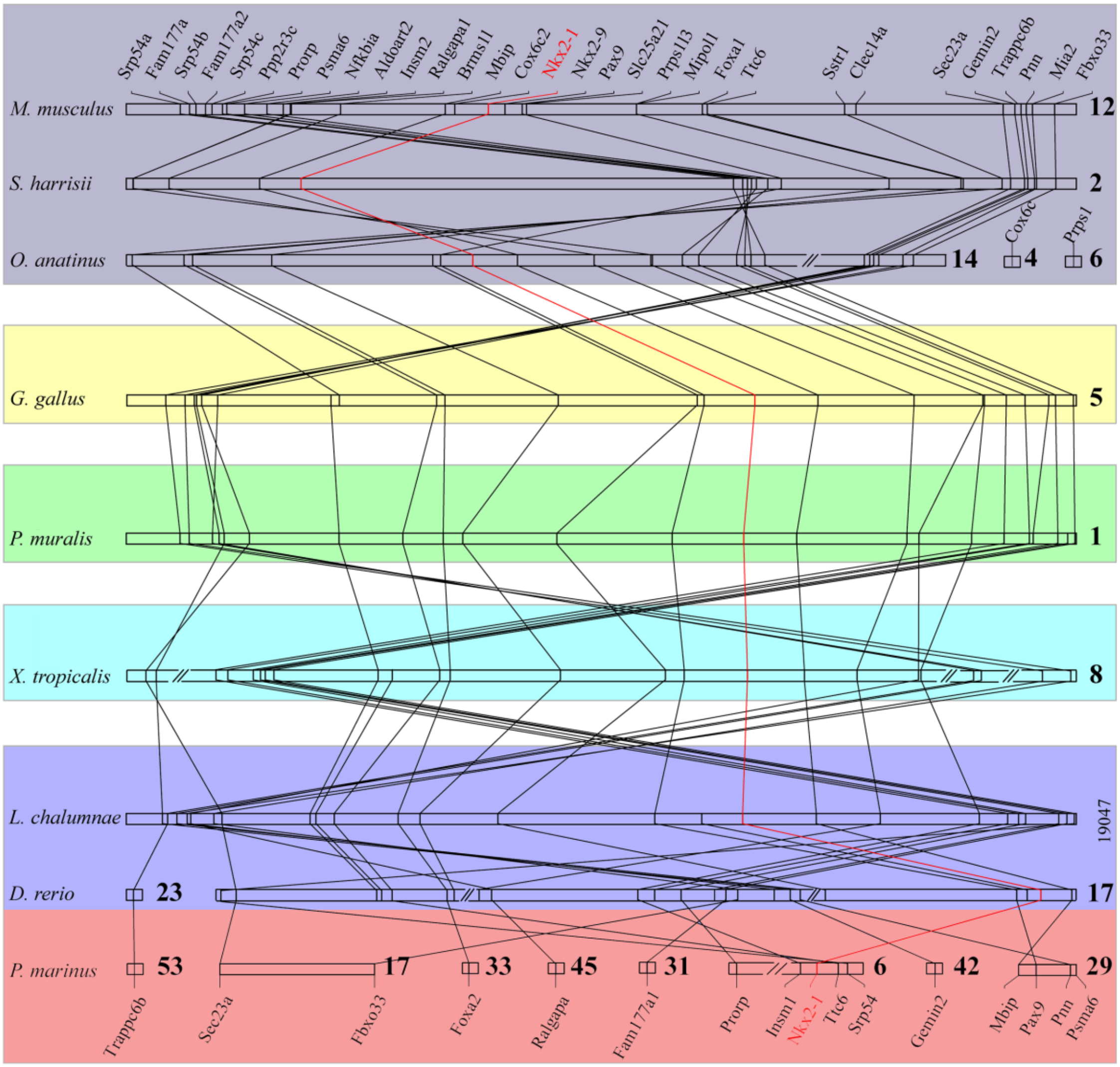

Fig. S9 Synteny of the mouse *Nkx2.1* (red) and its neighboring genes within 4.1 Mb chromosome (Chr) length. Numbers to the right of the box indicate Chr No. The compared species include monotreme (*Ornithorhynchus anatinus*), marsupial (*Sarcophilus harrisii)*, chick *(Gallus gallus)*, gecko *(Podarcis muralis)*, Xenopus *(Xenopus tropicalis)*, Latimeria (*Latimeria chalumnae)*, zebrafish *(Danio rerio)* and lamprey *(Petromyzon marinus)*. The chromosome localization maps were drawn by MapChart 2.32[1].

Fig. S10.

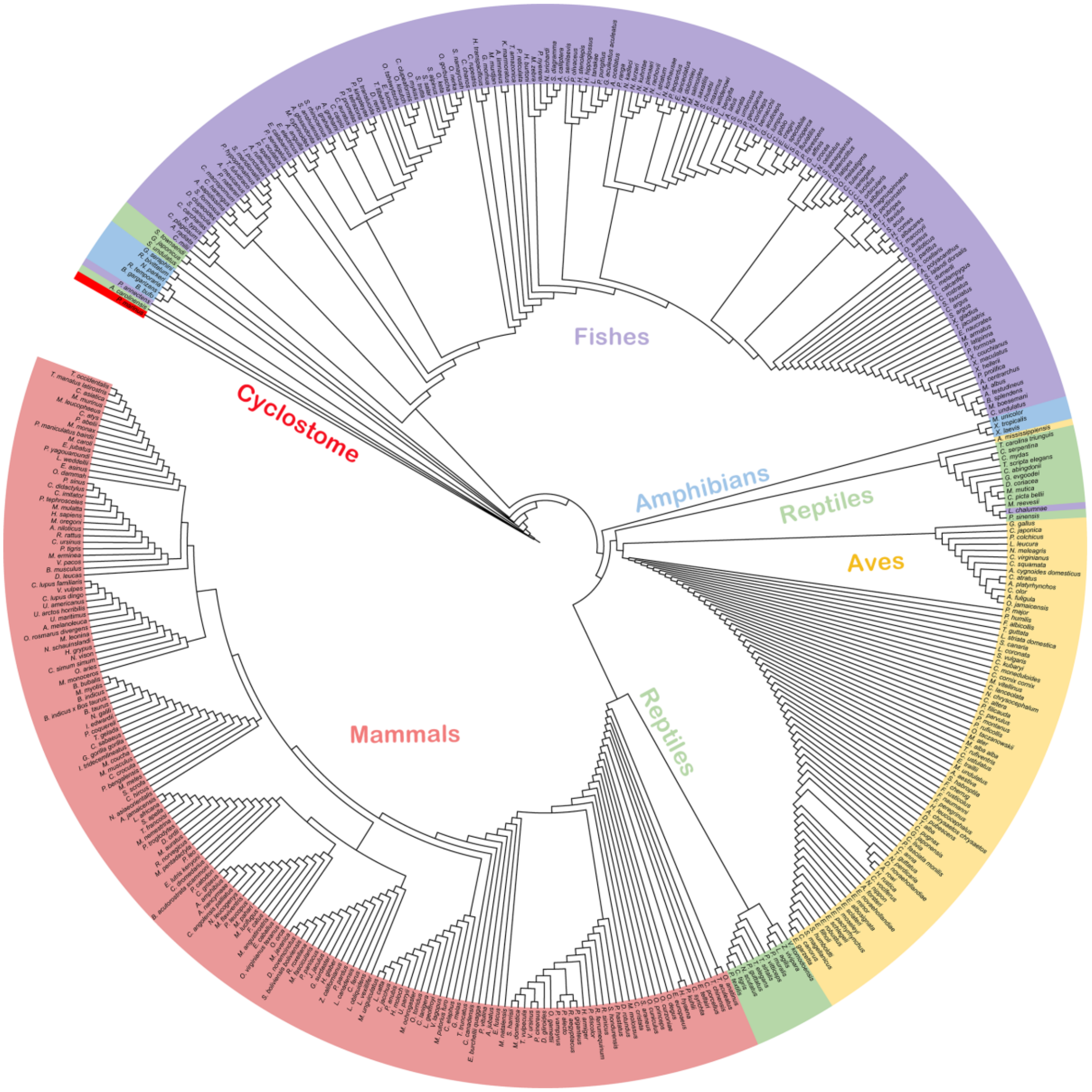

Fig. S10 Phylogenetic tree of Emx2 based on the neighbor-joining method. Neighbor-joining phylogenetic trees were then generated (Dayhoff Model) and displayed using MEGA11 with 1000 bootstraps[2].

Fig. S11.

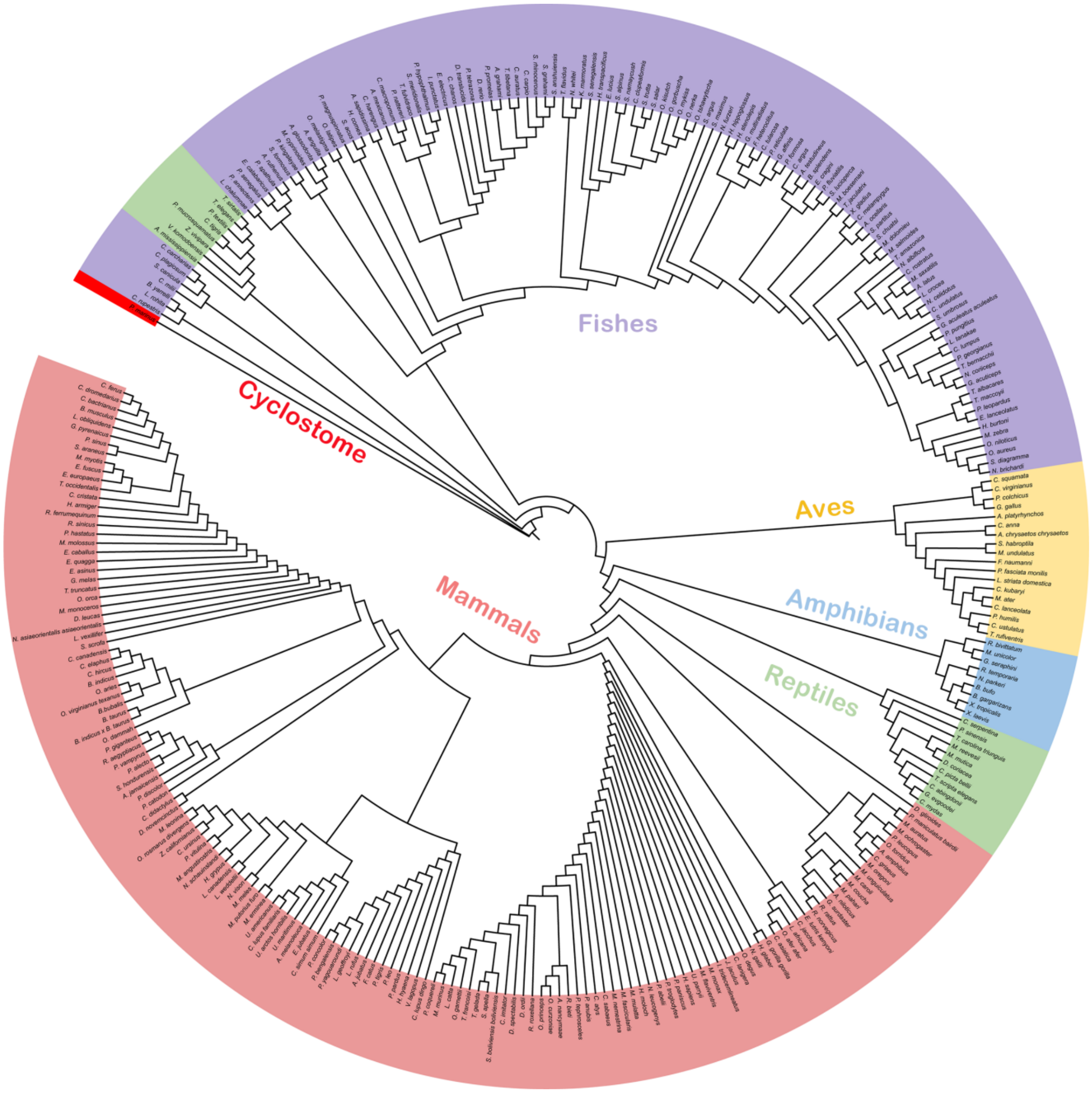

Fig. S11 Phylogenetic tree of Emx1 based on the neighbor-joining method. Neighbor-joining phylogenetic trees were then generated (Dayhoff Model) and displayed using MEGA11 with 1000 bootstraps[2].

Fig. S12.

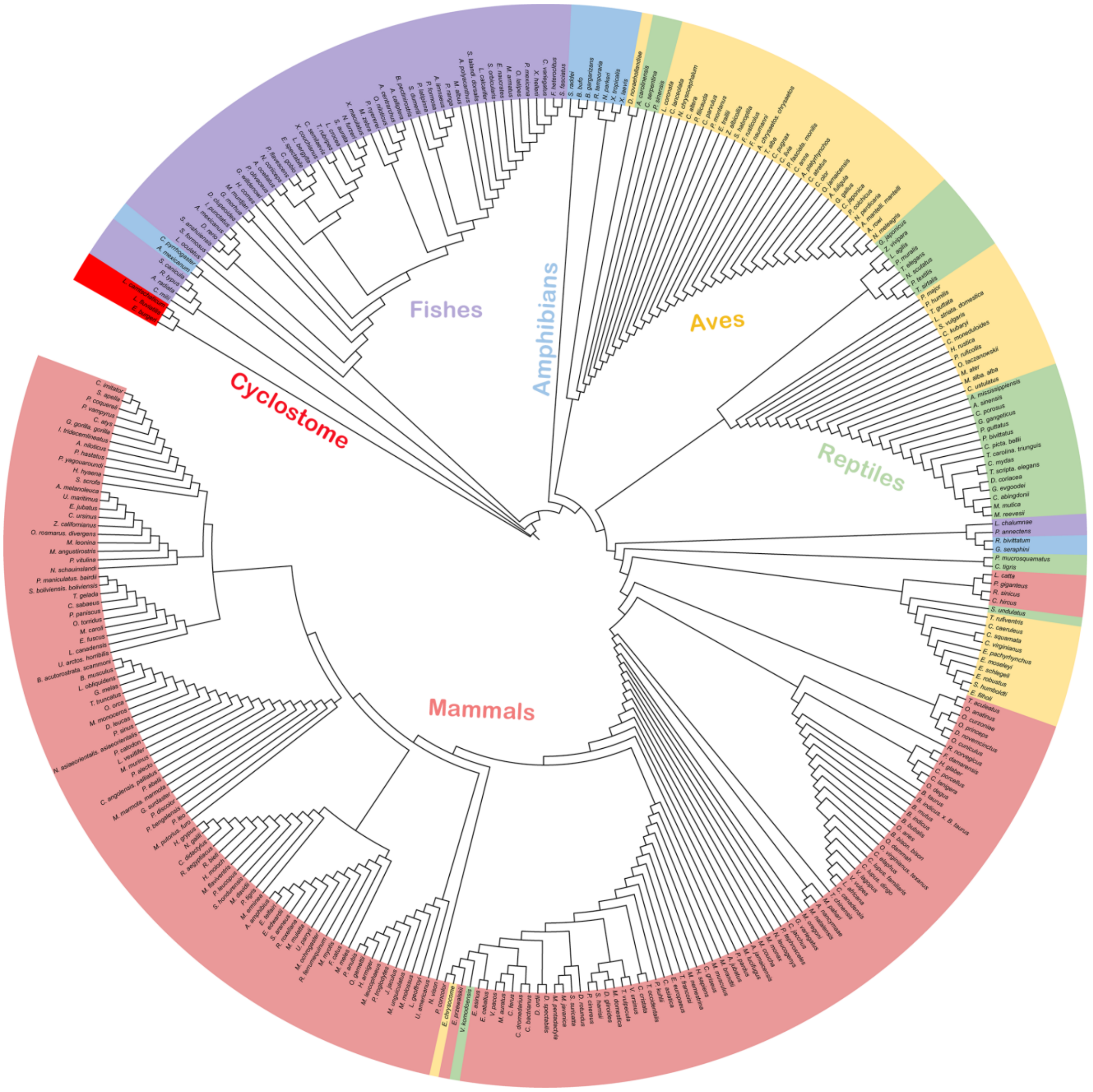

Fig. S12 Phylogenetic tree of Pax6 based on the neighbor-joining method. Neighbor-joining phylogenetic trees were then generated (Dayhoff Model) and displayed using MEGA11 with 1000 bootstraps[2].

Fig. S13.

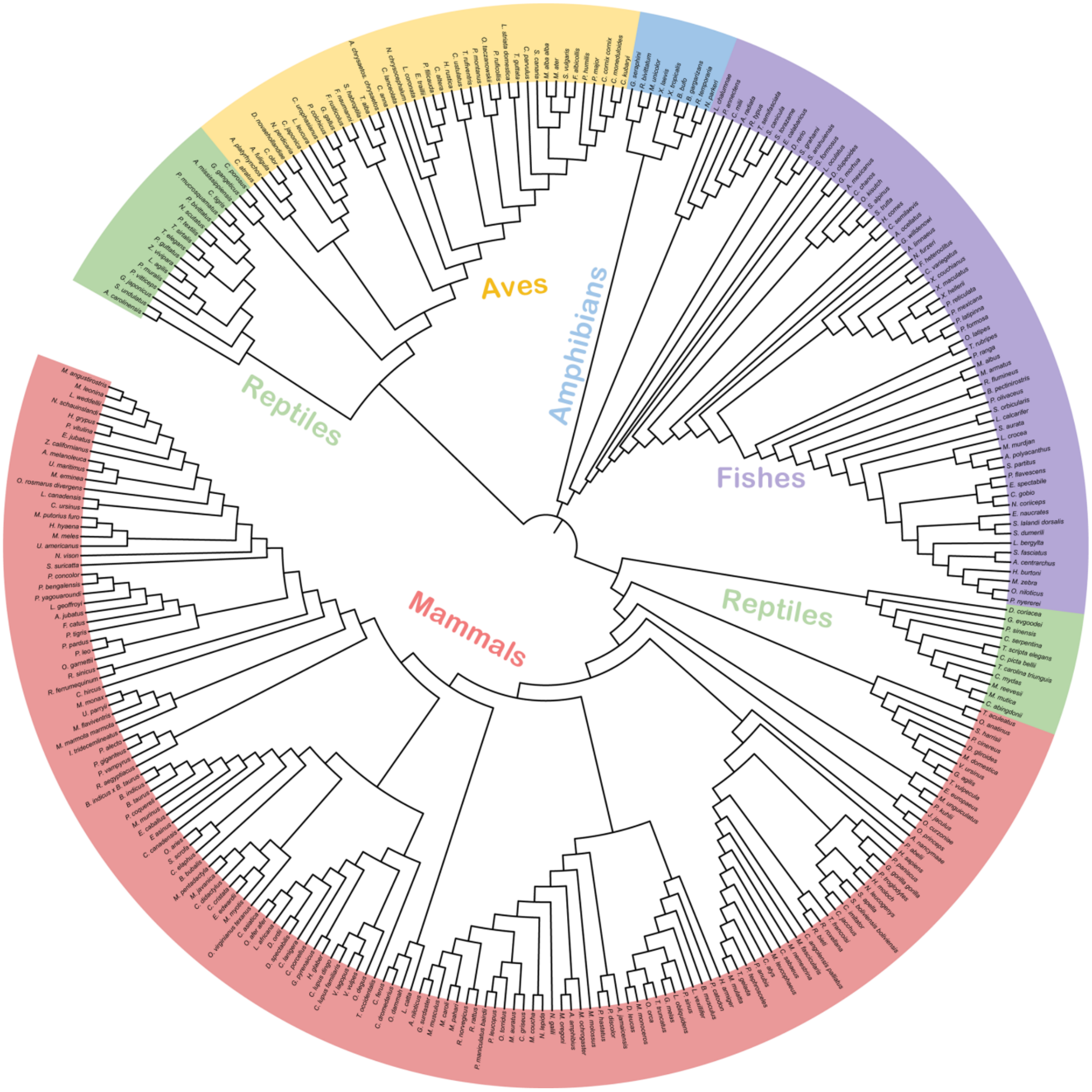

Fig. S13 Phylogenetic tree of Dlx2 based on the neighbor-joining method. Neighbor-joining phylogenetic trees were then generated (Dayhoff Model) and displayed using MEGA11 with 1000 bootstraps[2].

Fig. S14.

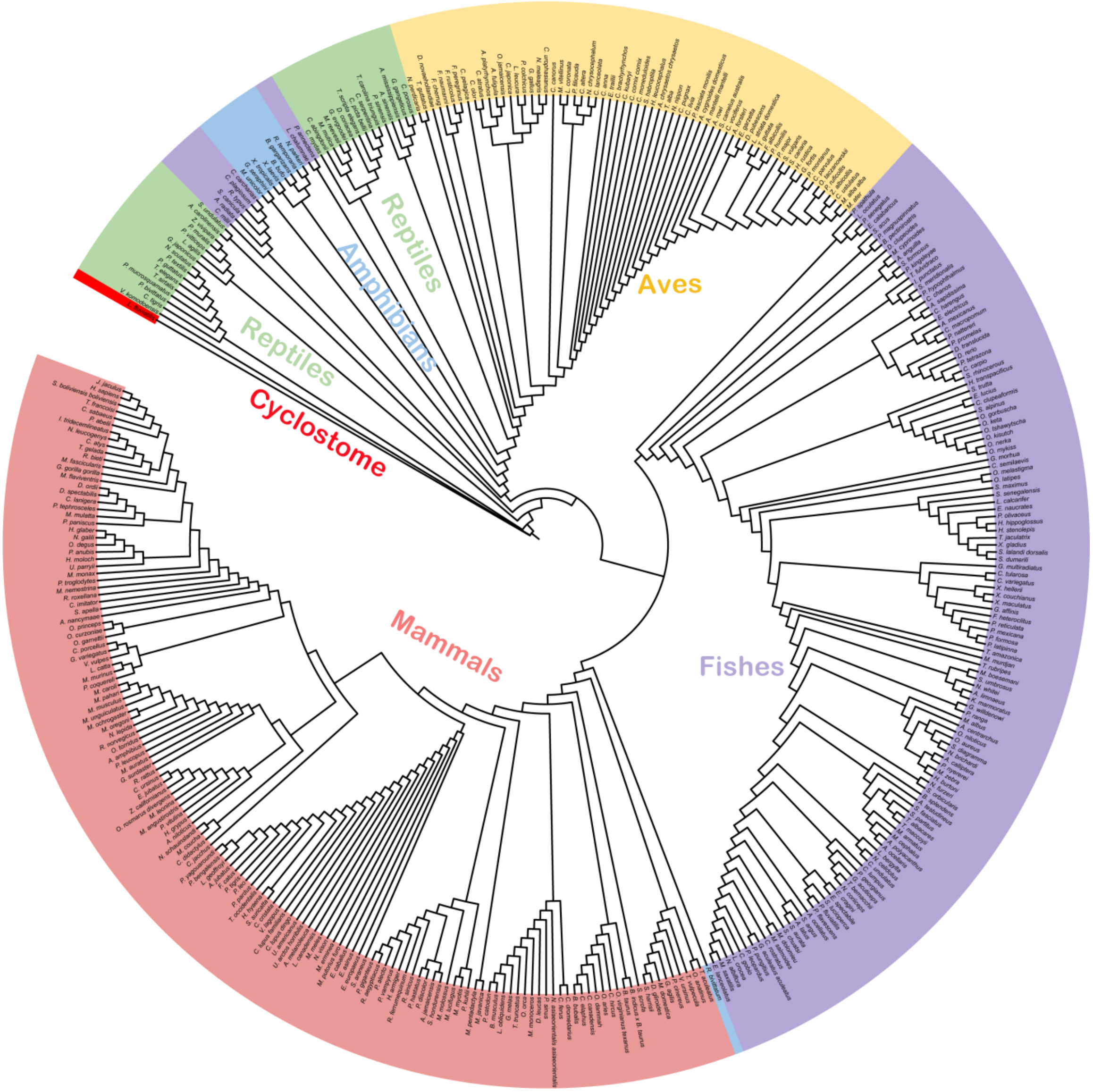

Fig. S14 Phylogenetic tree of Nkx2.1 based on the neighbor-joining method. Neighbor-joining phylogenetic trees were then generated (Dayhoff Model) and displayed using MEGA11 with 1000 bootstraps[2].

Fig. S15.

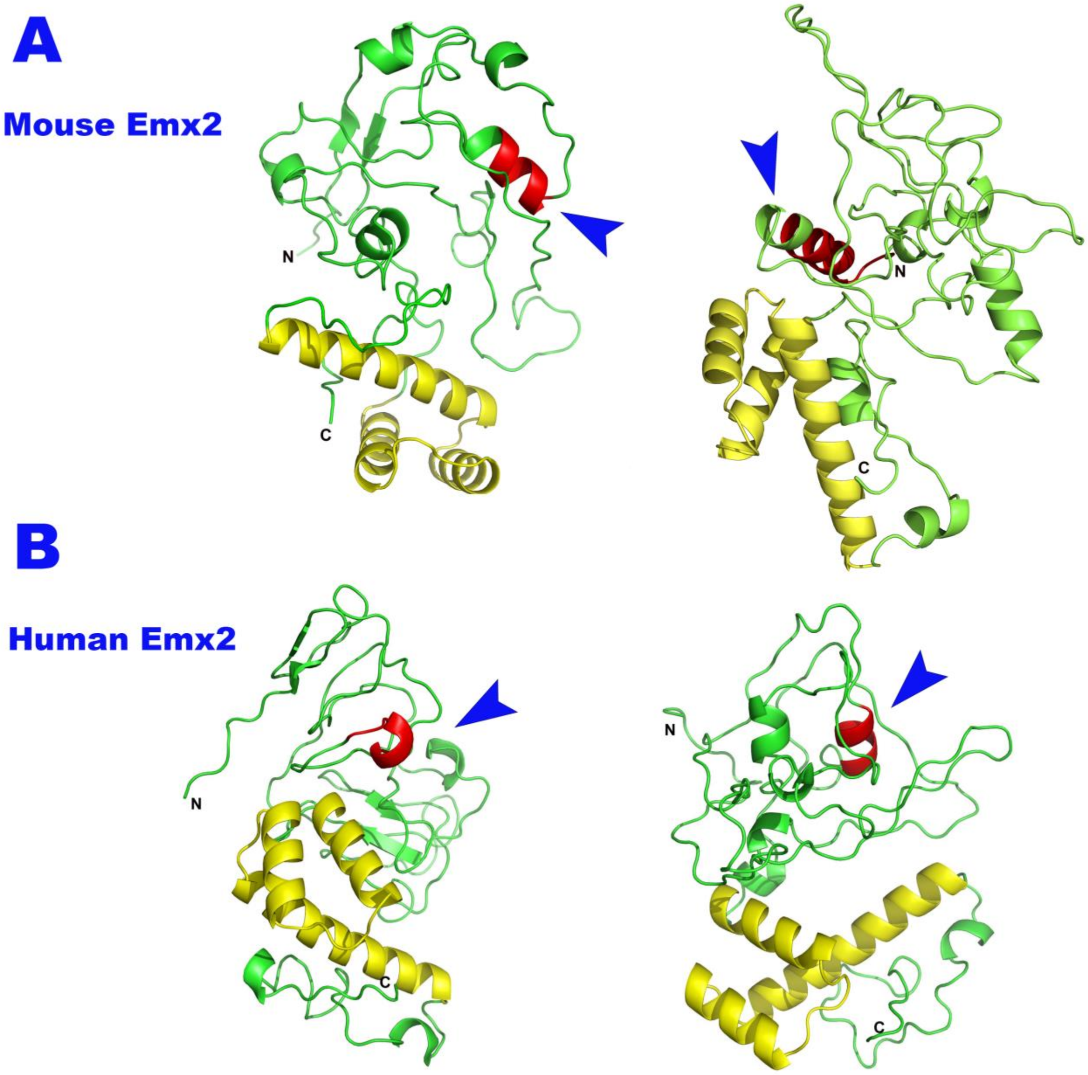

Fig. S15 Structural solutions of mouse and human Emx2 based on de novo structure prediction using Rosetta. A: mouse Emx2; B: human Emx2. The homeodomain is shown in yellow, and the alanine tract is shown in red (indicated by an arrowhead). The rest of the protein is shown in green.

Fig. S16.

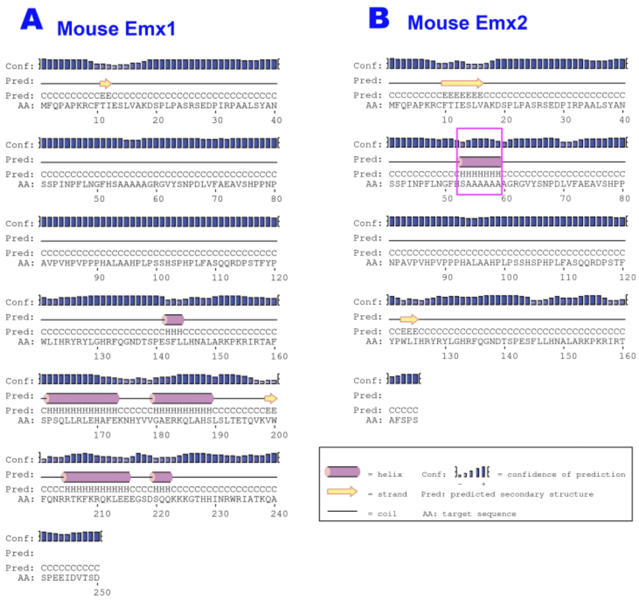

Fig. S16 Protein domain prediction using Dompred. (A) Mouse Emx1. (B) Mouse Emx2 with a helix formed by a tract of 7 Ala residues (marked by a red box).

Fig. S17.

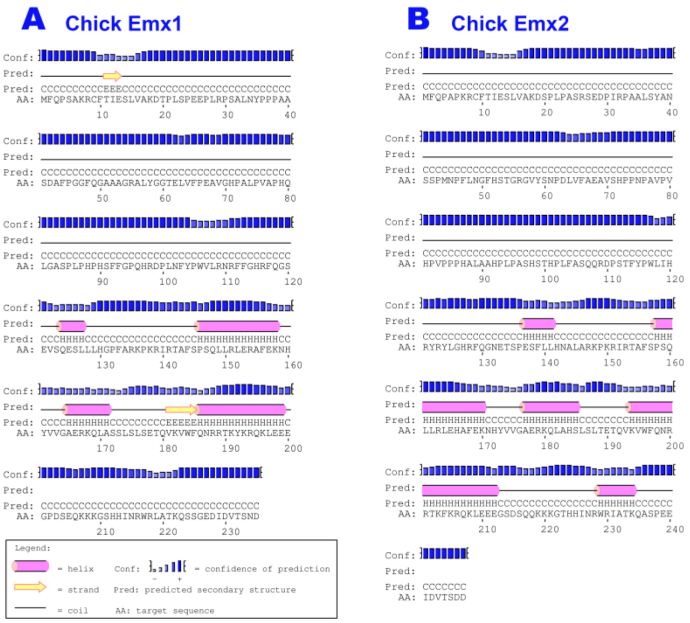

Fig. S17 Protein domain prediction using Dompred. (A) Chick Emx1. (B) Chick Emx2 without a helix formed by a tract of poly (Ala) residues.

Fig. S18.

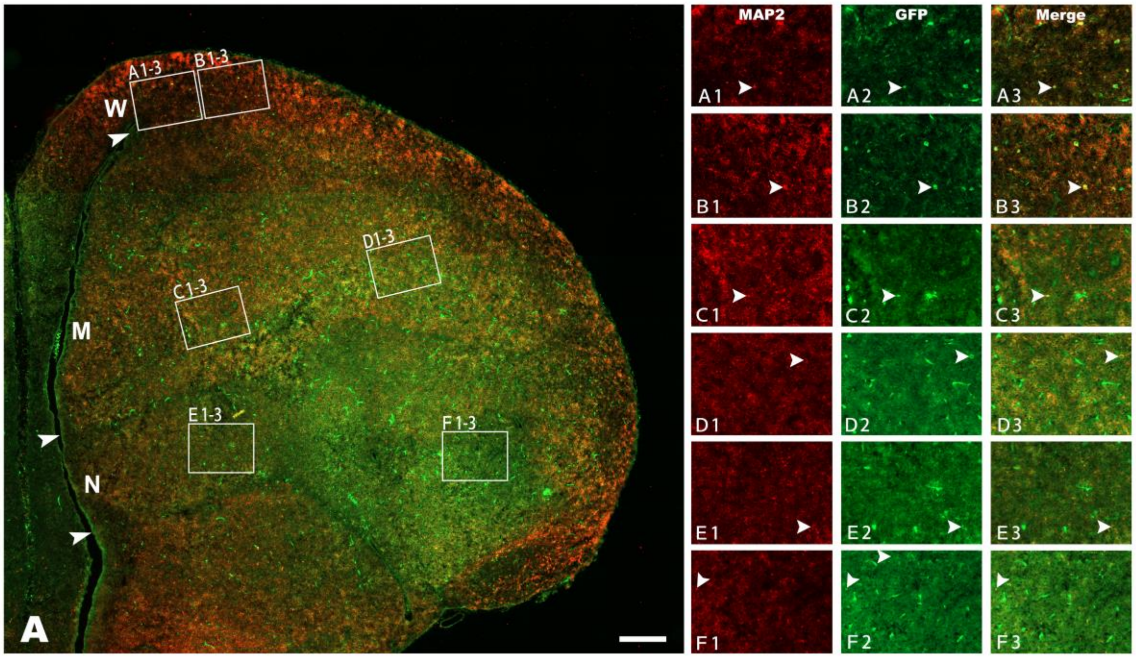

Fig. S18 MAP2 distribution in the pallium at embryonic day (E) 16 after injection of lentiviruses expressing mouse *Emx2* (*m-Emx2*) into the ventricle of the telencephalon at E3. The boundaries of Wulst (W), Mesopallium (M) and Nidopallium (N) are indicated by arrowheads. MAP2 expression levels in medial (A1-3, C1-3 and E1-3) and outside (B1-3, D1-3 and F1-3) areas are amplified in A1-F3, respectively. Some cells labeled for MAP2 (red) and infected with lentiviruses (expressing GFP) are indicated by arrowheads. Scale bar =200 µm.

Fig. S19.

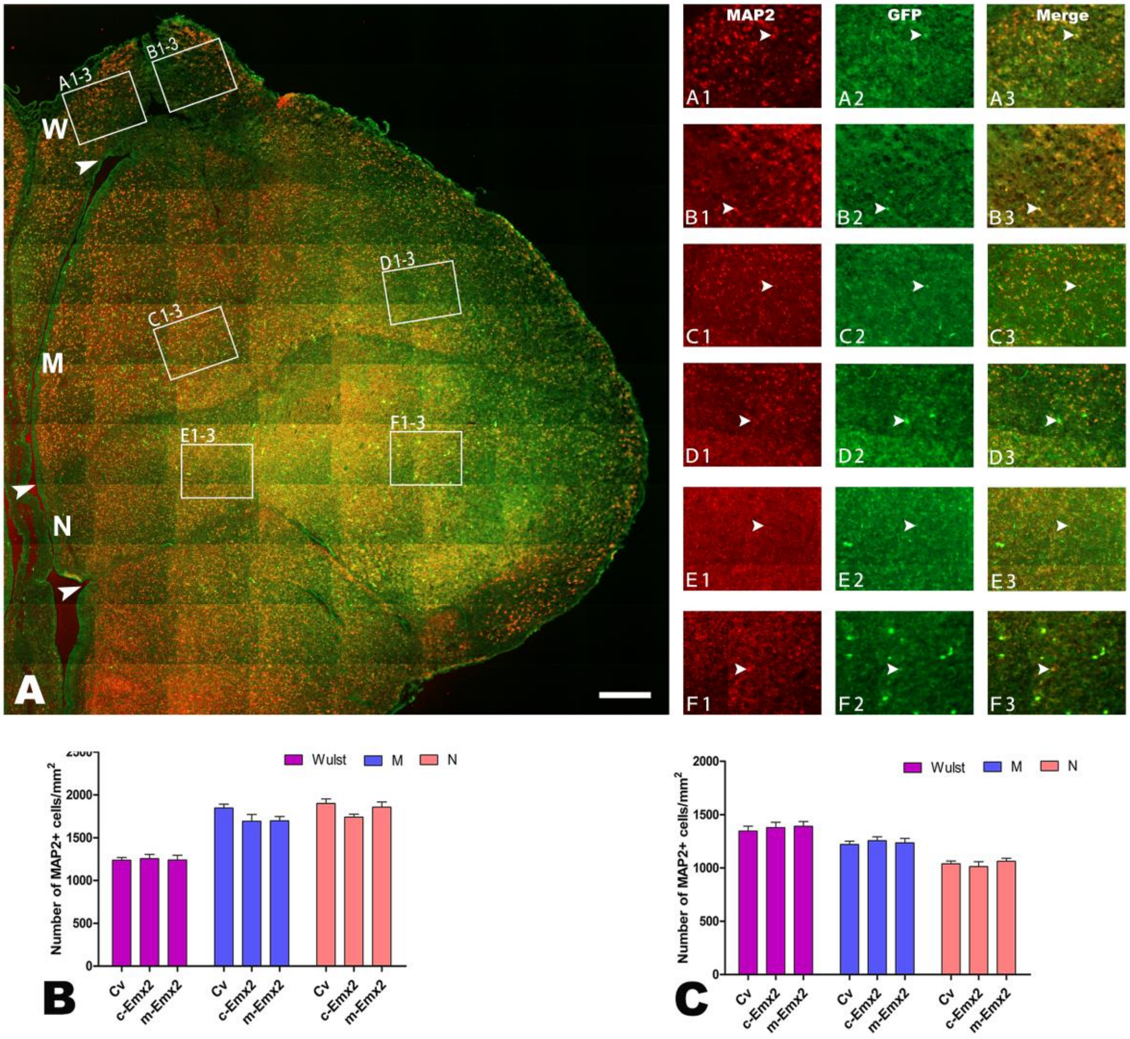

Fig. S19 MAP2 distribution in the pallium at embryonic day (E) 16 after injection of lentiviruses expressing chick *Emx2* (*c-Emx2*) into the ventricle of the telencephalon at E3. The boundaries of W, M and N are indicated by arrowheads. MAP2 expression levels in medial (A1-3, C1-3 and E1-3) and outside (B1-3, D1-3 and F1-3) areas are amplified in A1-F3, respectively. Some cells labeled for MAP2 (red) and infected with lentiviruses (expressing GFP) are indicated by arrowheads. B and C： Comparison of the numbers of MAP2 cells per mm^2 in the medial (B) and outside (C) regions of W, M and N among the groups after injections of control lentiviruses (Cv) and *m-Emx2* or *c-Emx2* lentiviruses. Scale bar =200 µm. *p<0.05 (SPSS, one-way ANOVA).

Fig. S20.

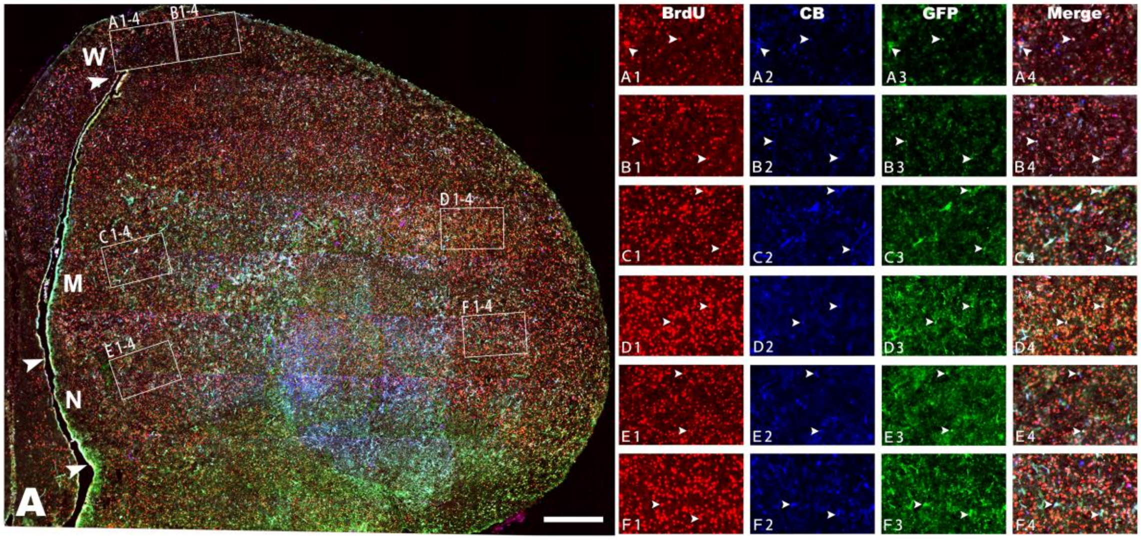

Fig. S20 Labeling for BrdU and calbindin (CB) in the Wulst (W), Mesopallium (M) and Nidopallium (N) after injection of lentiviruses expressing mouse *Emx2* into the ventricle of the telencephalon at embryonic Day 3 (E3). To label proliferating cells, a single dose of BrdU was injected into the hatching eggs at E6. The boundaries of W, M and N are indicated by arrowheads. BrdU and CB expression levels in inside (A1-4, C1-4 and E1-4) and outside (B1-4, D1-4 and F1-4) areas are amplified in A1-F4, respectively. Some double-labeled cells for BrdU (red) and CB (blue) and infected with lentiviruses (expressing GFP, green) are indicated by arrowheads. Scale bar =200 µm.

Fig. S21.

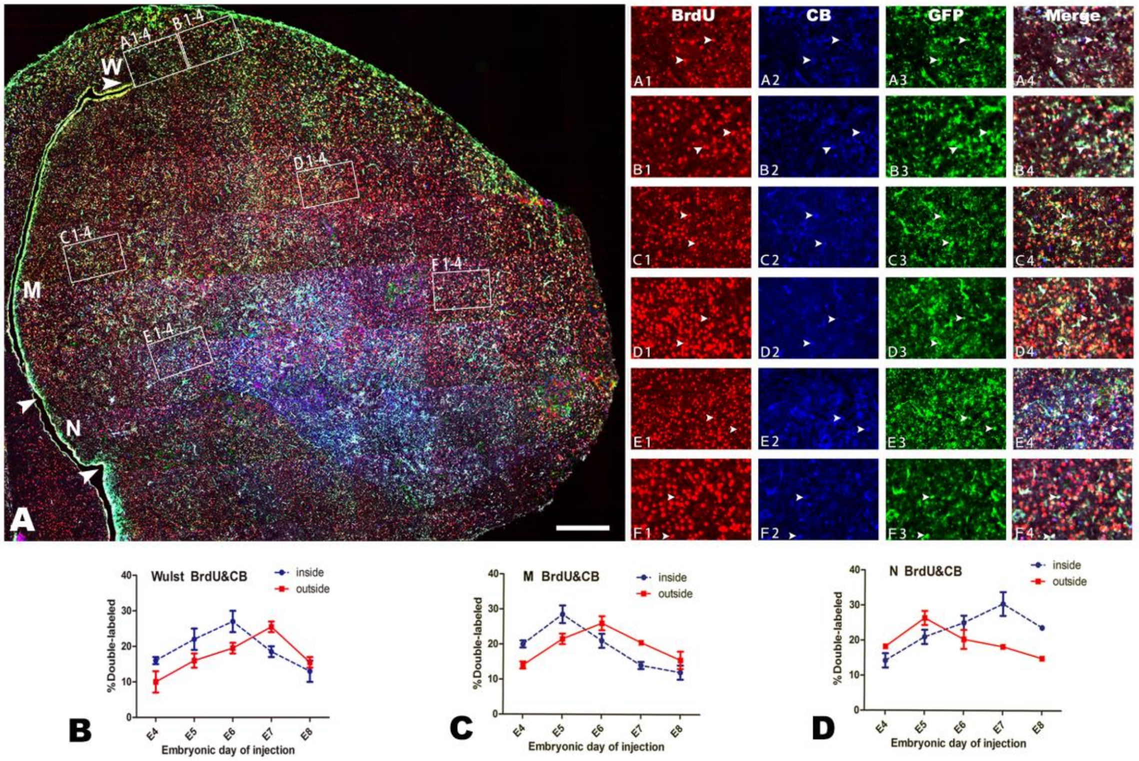

Fig. S21 Labeling for BrdU and calbindin (CB) in the Wulst (W), Mesopallium (M) and Nidopallium (N) after injection of lentiviruses expressing mouse *Emx2* into the ventricle of the telencephalon at embryonic Day 3 (E3). To label proliferating cells, a single dose of BrdU was injected into the hatching eggs at E7. The boundaries of W, M and N are indicated by arrowheads. BrdU and CB expression levels in inside (A1-4, C1-4 and E1-4) and outside (B1-4, D1-4 and F1-4) areas are amplified in A1-F4, respectively. Some double-labeled cells for BrdU (red) and CB (blue) and infected with lentiviruses (expressing GFP, green) are indicated by arrowheads. Scale bar in B=200 μm. B-D: Histograms illustrate neurogenesis gradients of BrdU-CB doubled cells in the W (C), M (D) and N (E) after BrdU injection into the embryos at different ages. Note that BrdU-CB double-labeled cells follow the "inside-out" neurogenesis gradient in W and M but the "inside-out" gradient in N.

Fig. S22.

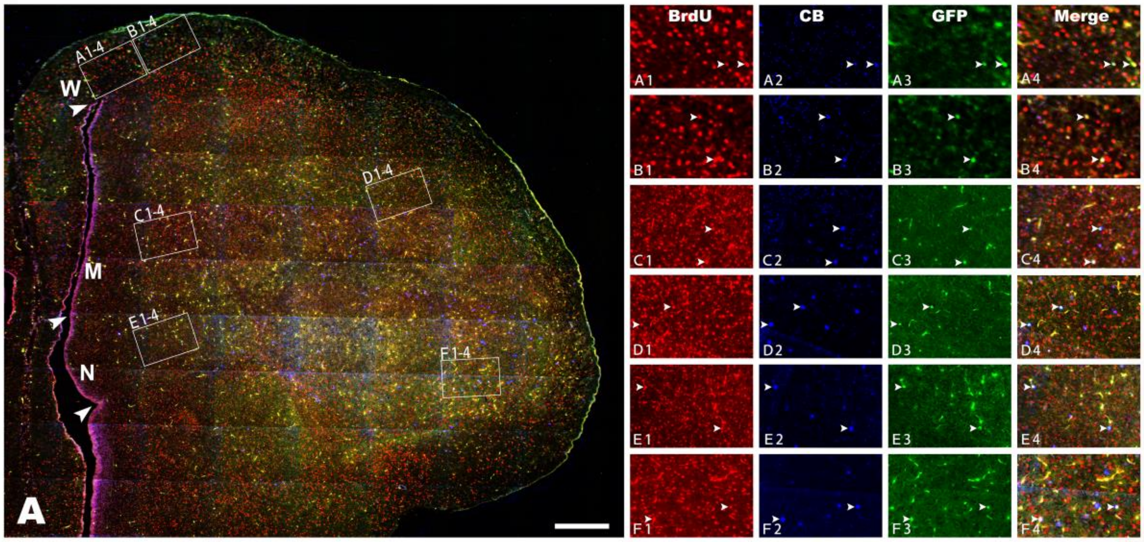

Fig. S22 Labeling for BrdU and calbindin (CB) in the Wulst (W), Mesopallium (M) and Nidopallium (N) after injection of lentiviruses expressing chick *Emx2* into the ventricle of the telencephalon at embryonic Day 3 (E3). To label proliferating cells, a single dose of BrdU was injected into the hatching eggs at E5. The boundaries of W, M and N are indicated by arrowheads. BrdU and CB expression levels in inside (A1-4, C1-4 and E1-4) and outside (B1-4, D1-4 and F1-4) areas are amplified in A1-F4, respectively. Some double-labeled cells for BrdU (red) and CB (blue) and infected with lentiviruses (expressing GFP, green) are indicated by arrowheads. Scale bar =200 μm.

Fig. S23.

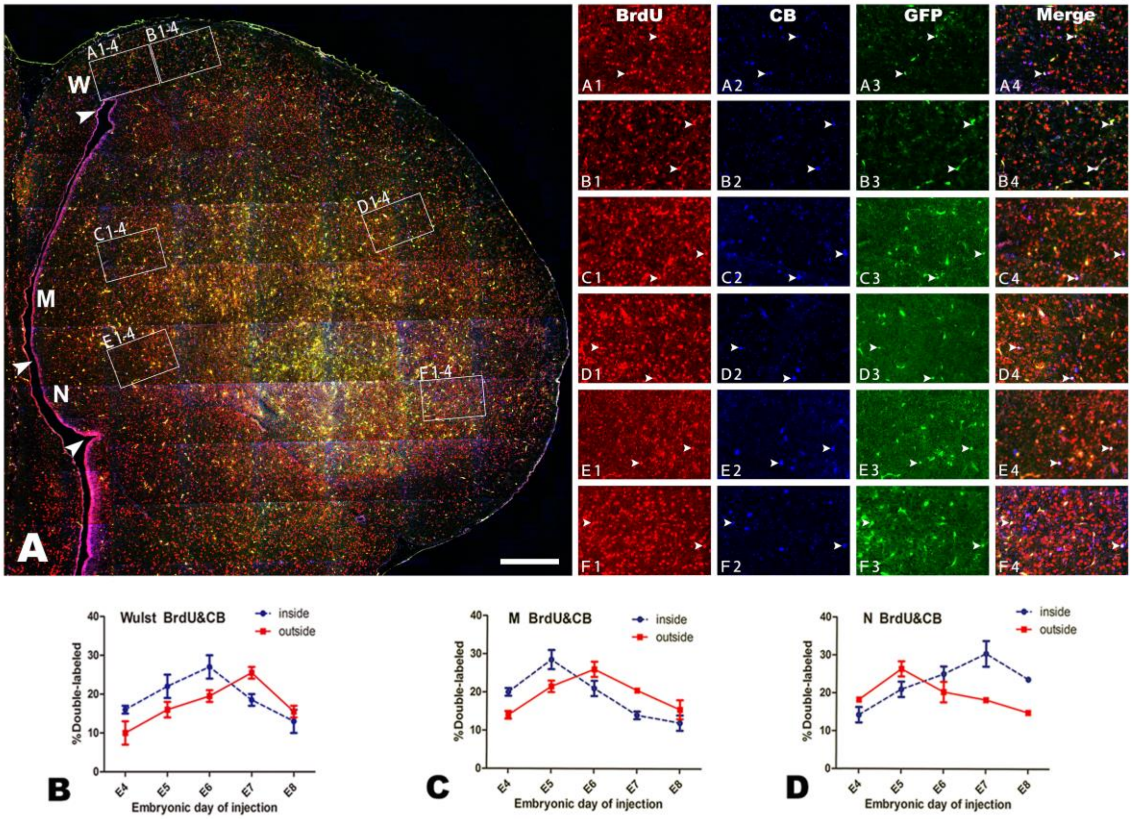

Fig. S23 Labeling for BrdU and calbindin (CB) in the Wulst (W), Mesopallium (M) and Nidopallium (N) after injection of lentiviruses expressing chick *Emx2* into the ventricle of the telencephalon at embryonic Day 3 (E3). To label proliferating cells, a single dose of BrdU was injected into the hatching eggs at E6. The boundaries of W, M and N are indicated by arrowheads. BrdU and CB expression levels in inside (A1-4, C1-4 and E1-4) and outside (B1-4, D1-4 and F1-4) areas are amplified in A1-F4, respectively. Some double-labeled cells for BrdU (red) and CB (blue) and infected with lentiviruses (expressing GFP, green) are indicated by arrowheads. Scale bar in B=200 µm. B-D: Histograms illustrate neurogenesis gradients of BrdU-CB doubled cells in the W (C), M (D) and N (E) after BrdU injection into the embryos at different ages.

Fig. S24.

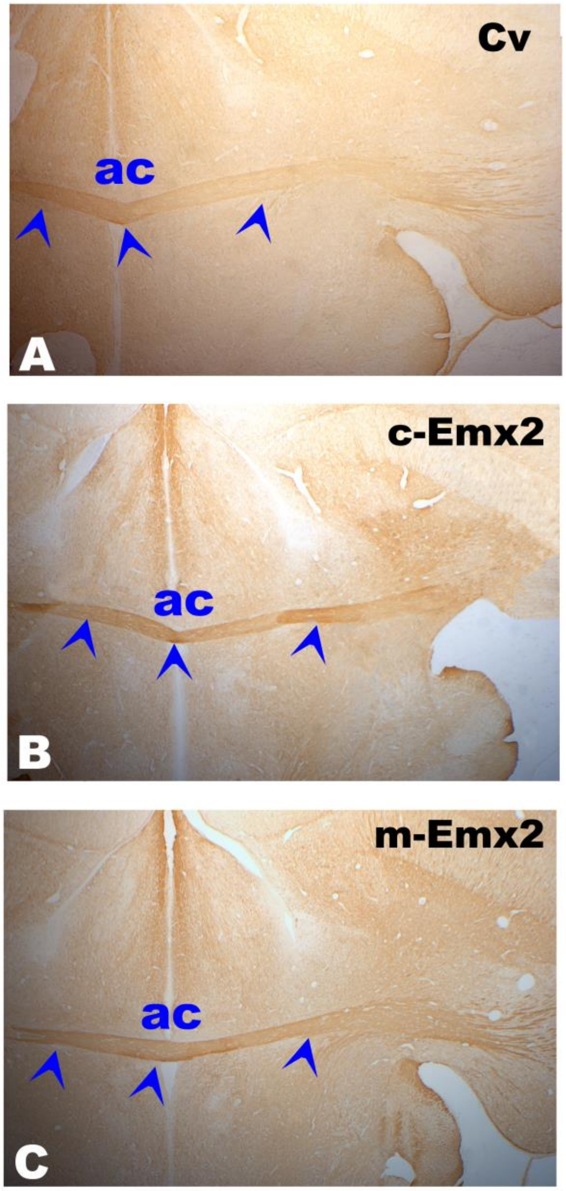

Fig. S24 Anterior commissure (ac) labeled for neurofilaments in the telencephalon of chicks at the age of 9 days. (A-C) The ac in the telencephalon of chicks after injection of control (Cv, A), *m-Emx2* (B) or *c-Emx2* (C) lentiviruses into the ventricle at embryonic Day 3. Scale bar in C=500 µm for A-C.

Fig. S25.

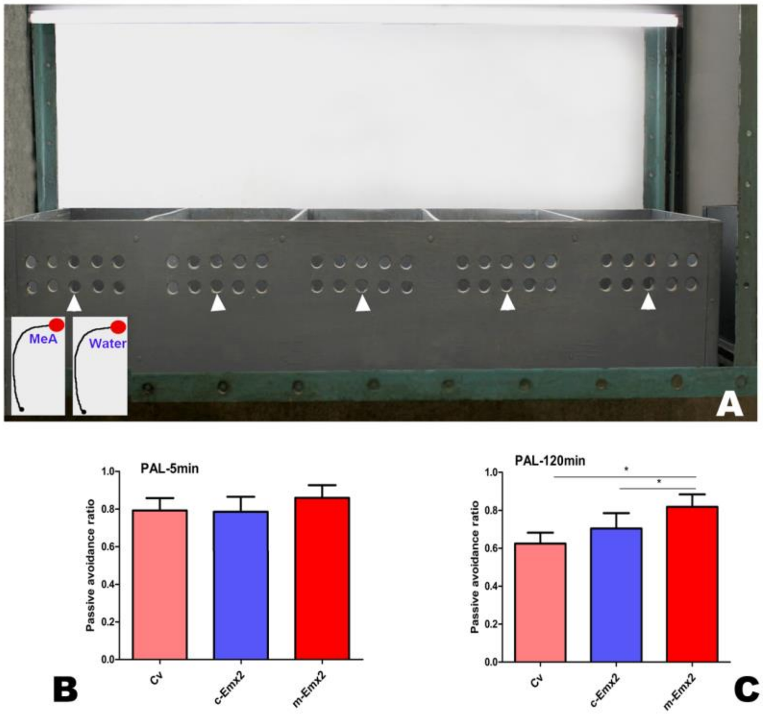

Fig. S25 Test for the one-trial passive avoidance learning task. (A) The equipment used in the one-trial passive avoidance learning (PAL) task. Chicks at the age of 1 day were placed into the boxes in pairs. Beads coated with pure water or coated with methyl anthranilate (MeA) were presented to chicks through small holes in the box (arrowheads). Chicks that pecked the MeA bead and evinced a disgust response were recorded. For the details of the test, see the text. (B and C) Passive avoidance ratio: background numbers (BN) / (BN + pecking numbers) tested at 5 min (B) and 12 min (C) are compared among the groups receiving injection of control lentiviruses (Cv), *m-Emx2* or *c-Emx2* lentiviruses into the ventricle at embryonic Day 3. *p<0.05 (SPSS, one-way ANOVA).

Fig. S26.

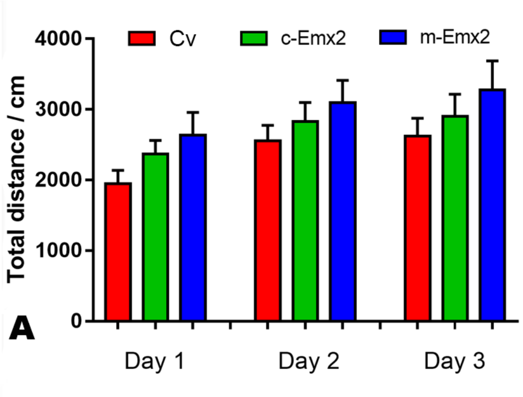

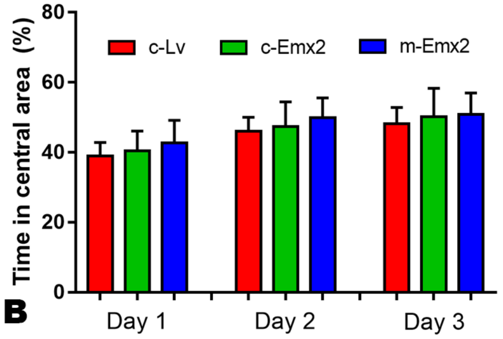

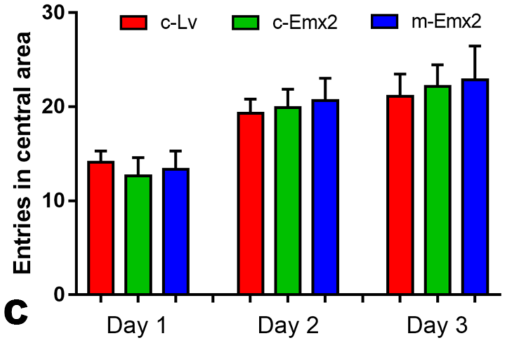

Fig. S26 Comparison of locomotor activities in the open-field test. There were no significant differences among the groups receiving injection of control lentiviruses (cLv), *m-Emx2* or *c-Emx2* lentiviruses into the ventricle at embryonic Day 3 in the total distance (A), the percentage of time spent in the central rectangle (B) and the number of entries into the central area on three successive days. *p>0.05 (SPSS, one-way ANOVA).

Table S1.

The species and gene numbers examined in this study

		Emx1		Emx2		Pax6		Dlx2		Nkx2.1	
		**Species**	**No.**	**Species**	**No.**	**Species**	**No.**	**Species**	**No.**	**Species**	**No.**
**Mammals**	Eutheria	179	312	179	480	171	2233	170	220	158	453
	Metatheria	6	9	7	12	7	15	7	7	7	7
	Prototheria	/	/	2	4	2	12	2	2	2	2
	Total	185	321	188	496	180	2260	179	229	167	462
**Birds**		64	75	93	180	111	797	54	84	80	104
**Reptiles**		27	29	32	58	34	311	31	34	32	36
**Amphibians**		9	18	10	16	14	82	10	12	9	13
**Fish**	coelacanths	1	1	1	1	1	2	1	1	1	1
	lungfish	1	1	1	1	1	2	1	1	1	1
	bony fishes	117	209	156	240	66	448	337	780	134	174
	cartilaginous fishes	4	7	6	9	5	42	16	16	6	7
	Total	123	218	164	251	73	494	355	798	142	183
**Cyclostome**		1	1#	1	1##	3	4	/	/	1	1
	Total	409	662	488	1002	415	3948	629	1157	431	799
**Chromosomal locus**			1*		1*		1*		1		1

No. Numbers of the genes with different versions.

#Emx A, ##Emx B. *Some exceptions are shown in the text.

Table S2.

The numbers of chromosomes in which 30 genes adjacent to the examined homeobox genes in the mouse were located

		Emx1	Emx2	Pax6	Dlx	Nkx2
	Chromosome length (Mb)	2.7	3.8	6.8	3.7	4.1
Mammals	*Mus musculus*	1	1	1	1	1
	Sarcophilus harrisii	2	2	1	1	1
	Ornithorhynchus anatinus	3	2	1	1	3
Bird	*Gallus gallus*	5	2	1	1	1
Reptile	*Podarcis muralis*	3	2	1	1	1
Amphabia	*Xenopus tropicalis*	3	2	1	1	1
Fish	*Latimeria chalumnae*	9	6	4	9	1
	Danio rerio	9	6	3	4	2
cyclostome	*Petromyzon marinus*	8	4	6	4	8

Table S3.

The species used for studying Darwinian selection by Ka/Ks ratios

Class	subclass/*Order*	*Specieces*
Mammals (20)	Eutheria	*Ochotona princeps, Vombatus ursinus, Ursus arctos horribilis, Mus musculus, Homo sapiens, Odobenus rosmarus divergens, Macaca mulatta, Acinonyx jubatus, Phascolarctos cinereus, Monodelphis domestica, Tursiops truncates, Bos Taurus, Mustela putorius furo, Canis lupus familiaris, Castor canadensis, Pan troglodytes*
	Metetheria	*Sarcophilus harrisii*
	Prototheria	*Ornithorhynchus anatinus*
Birds (20)		*Lepidothrix coronate, Taeniopygia guttata, Serinus canaria, Empidonax traillii, Cygnus atratus, Corvus cornix cornix, Phasianus colchicus, Sturnus vulgaris, Aquila chrysaetos, Motacilla alba alba, Pseudopodoces humilis, Tyto alba, Lonchura striata domestica*
Reptiles (20)	Crocodylia	*Gavialis gangeticus, Pelodiscus sinensis, Chelonia mydas, Alligator mississippiensis , Terrapene carolina triunguis*
	Testudines	*Dermochelys coriacea, Mauremys reevesii, Podarcis muralis, Gopherus evgoodei, Sceloporus undulates, Gekko japonicus, Chrysemys picta, Trachemys scripta elegans*
	Lepidosauria	*Thamnophis elegans, Pantherophis guttatus, Lacerta agilis, Thamnophis sirtalis, Notechis scutatus, Zootoca vivipara, Pseudonaja textilis*
Amphibians (7)		*Nanorana parkeri, Rana temporaria, Xenopus tropicalis, Rhinatrema bivittatum, Geotrypetes seraphini, Bufo bufo, Microcaecilia unicolor*
Fishes (24)	Coelacanthiformes	*Latimeria chalumnae*
	Actinopterygii	*Oryzias melastigma, Maylandia zebra, Nothobranchius furzeri, Cynoglossus semilaevis, Lates calcarifer, Pundamilia nyererei, Archocentrus centrarchus, Oryzias latipes, Poecilia Formosa, Oreochromis aureus, Lepisosteus oculatus, Gadus morhua, Haplochromis burtoni, Astyanax mexicanus, Danio rerio, Neolamprologus brichardi, Oreochromis niloticus, Cyprinodon variegatus, Astatotilapia calliptera*
	Chondrichthye	*Callorhinchus milii, Amblyraja radiata, Scyliorhinus canicular*
Cyclostome (1)	Petromyzontiforme	*Petromyzon marinus*

Movies S1 to S3.

Open-field tests for the chicks

Open-field tests for the chicks to assess their locomotor activity, curious and exploratory behaviors. The total distance of movement, the percentage of time spent in the central area and the number of entries into the central area were assessed. For each chick, the test was performed on three successive days (from posthatching day 5 to 7).

Movie S1：Open-field tests for the chicks treated with the control lentiviruses.

Movie S2：Open-field tests for the chicks treated with the lentiviruses expressing mouse Emx2.

Movie S3: Open-field tests for the chicks treated with the lentiviruses expressing chick Emx2.

SI References

1. Voorrips, R.E. MapChart: software for the graphical presentation of linkage maps and QTLs. *The J. Hered.* **93**, 77-78. https://doi.org/10.1093/jhered/93.1.77 (2002).

2. Kumar, S., Tamura, K., Jakobsen, I.B. & Nei, M. MEGA2: molecular evolutionary genetics analysis software. *Bioinformatics (Oxford, England)* **17**, 1244-1245. https://doi.org/10.1093/bioinformatics/17.12.1244 (2001).
